# Peripheral ablation of type III adenylyl cyclase induces hyperalgesia and eliminates KOR-mediated analgesia in mice

**DOI:** 10.1172/jci.insight.153191

**Published:** 2022-02-08

**Authors:** Wen-Wen Zhang, Hong Cao, Yang Li, Xian-Jun Fu, Yu-Qiu Zhang

**Affiliations:** 1 State Key Laboratory of Medical Neurobiology and MOE Frontiers Center for Brain Science, Department of Translational Neuroscience, Jing’an District Centre Hospital of Shanghai, Institutes of Brain Science, Fudan University, Shanghai, China.; 2 College of Intelligence and Information Engineering Shandong University of Traditional Chinese Medicine, Jinan, Shandong, China.; 3 Qingdao Academy of Chinese Medical Science, Shandong University of Traditional Chinese Medicine, Qingdao, Shandong, China.

**Keywords:** Neuroscience, Pain

## Abstract

Ca^2+^/calmodulin-stimulated group I adenylyl cyclase (AC) isoforms AC1 and AC8 have been involved in nociceptive processing and morphine responses. However, whether AC3, another member of group I ACs, is involved in nociceptive transmission and regulates opioid receptor signaling remains elusive. Here, we report that conditional KO of AC3 (*AC3* CKO) in L3 and L4 DRGs robustly facilitated the mouse nociceptive responses, decreased voltage-gated potassium (Kv) channel currents, and increased neuronal excitability. Furthermore, we report *AC3* CKO eliminated the analgesic effect of κ-opioid receptor (KOR) agonist and its inhibition on Kv channel by classical Gα_i/o_ signaling or nonclassical direct interaction of KOR and AC3 proteins. Interestingly, significantly upregulated AC1 level and cAMP concentration were detected in AC3-deficient DRGs. Inhibition of AC1 completely reversed cAMP upregulation, neuronal excitability enhancement, and nociceptive behavioral hypersensitivity in *AC3*-CKO mice. Our findings suggest a crucial role of peripheral AC3 in nociceptive modulation and KOR opioid analgesia.

## Graphical abstract

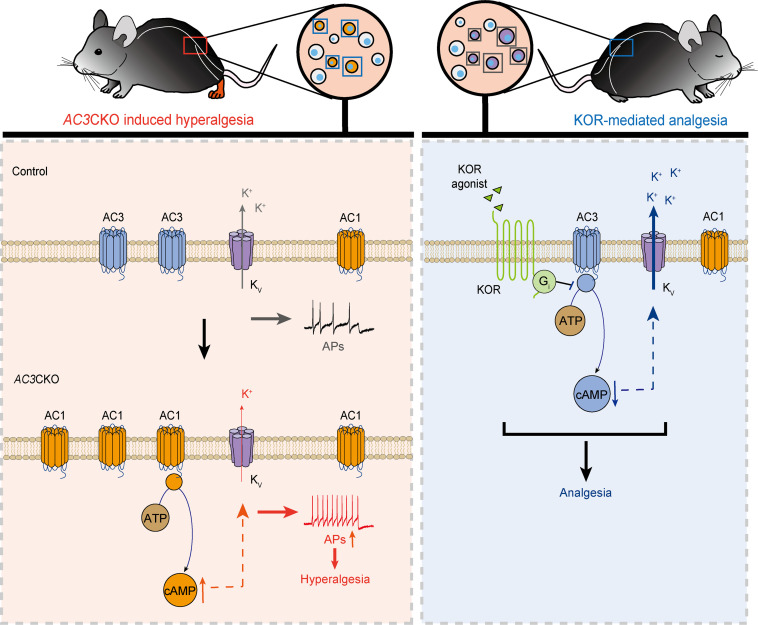

## Introduction

Mammalian adenylyl cyclase (AC) is an important enzyme family catalyzing the conversion of ATP into the second messenger cAMP in GPCR signaling. So far, 10 AC isoforms including 9 membrane-bound ACs (mAC, AC1–AC9) and 1 soluble AC (sAC, AC10) have been identified successively in mammals (1). Studies have demonstrated that AC isoforms are expressed ubiquitously in distinct regions and subcellular locations of the CNS and contribute to various pathophysiological processes, including learning, memory, anxiety, depression, chronic pain, and drug abuse (2–7). Among these AC isoforms, AC1, AC3, and AC8 can be directly stimulated by Ca^2+^/calmodulin in vitro and are classified into the Group I AC isoforms (8–11). AC1 and AC8 are highly expressed in the brain, such as hippocampus (12) and anterior cingulate cortex (13). AC1-KO and AC1/AC8–double KO (AC1/AC8-DKO) mice exhibit robust reduced formalin and CFA inflammatory pain responses (14–18). Pharmacological inhibition of AC1 can also effectively relieve inflammatory and neuropathic pain in both mice and rats (17, 19, 20). However, whether AC3, another member of Group I ACs, is implicated in nociceptive processing remains unclear.

AC3 was first identified in rat olfactory epithelium, residing in the olfactory neuronal cilia, which project into the nasal lumen and are accessible to airborne odorants (2). Later studies found that AC3 localizes to primary cilia on neurons, choroid plexus cells, and glial cells throughout the adult mouse brain (21, 22) and plays critical roles in learning, memory, sleep, anxiety, and depression (5, 7, 23, 24). Our recent study demonstrated that, in the dorsal root ganglion (DRG), AC3 was predominantly expressed in the soma of CGRP^+^ (a peptidergic nociceptor marker) sensory neurons, implying the involvement of AC3 in pain modulation (25). ACs, as downstream effectors for various GPCRs, can be activated by the Gα_s_ protein or can be inhibited by the Gα_i_ (26, 27). Opioids, adenosine and cannabinoids produce analgesic effects in nociceptors through activating Gα_i_ protein–coupled receptors that inhibit AC-mediated cAMP production (28). AC1- and AC8-induced cAMP signaling has been confirmed to be inhibited by morphine through Gα_i_ protein by coupling the μ-opioid receptor (18, 29), but little is known of AC3 in opioid-mediated signaling.

In the present study, employing Cre-loxp system, we conditionally knocked out AC3 (*AC3* CKO) in L3 and L4 DRGs by intra-DRG injection of Cre recombinase expressing adeno-associated virus in *AC3^fl/fl^* mice. In the *AC3*-CKO mice, we investigated whether and how the peripheral AC3 regulates nociceptive transmission, and we also assessed the role of AC3 in the opioid analgesia. Our findings demonstrate that peripheral KO of AC3 resulted in significantly nociceptive behavioral hypersensitivity, attenuated voltage-gated potassium (Kv) channel currents, and increased neuronal excitability in DRG neurons. Moreover, we found that AC3 in DRGs was involved in the analgesic effect mediated by KOR, but not MOR and DOR. Our results uncover an emerging role for peripheral AC3 signaling in KOR analgesia and nociceptive modulation.

## Results

### Selective KO of AC3 in DRG neurons induces hyperalgesia in mice.

**Figure 1:**
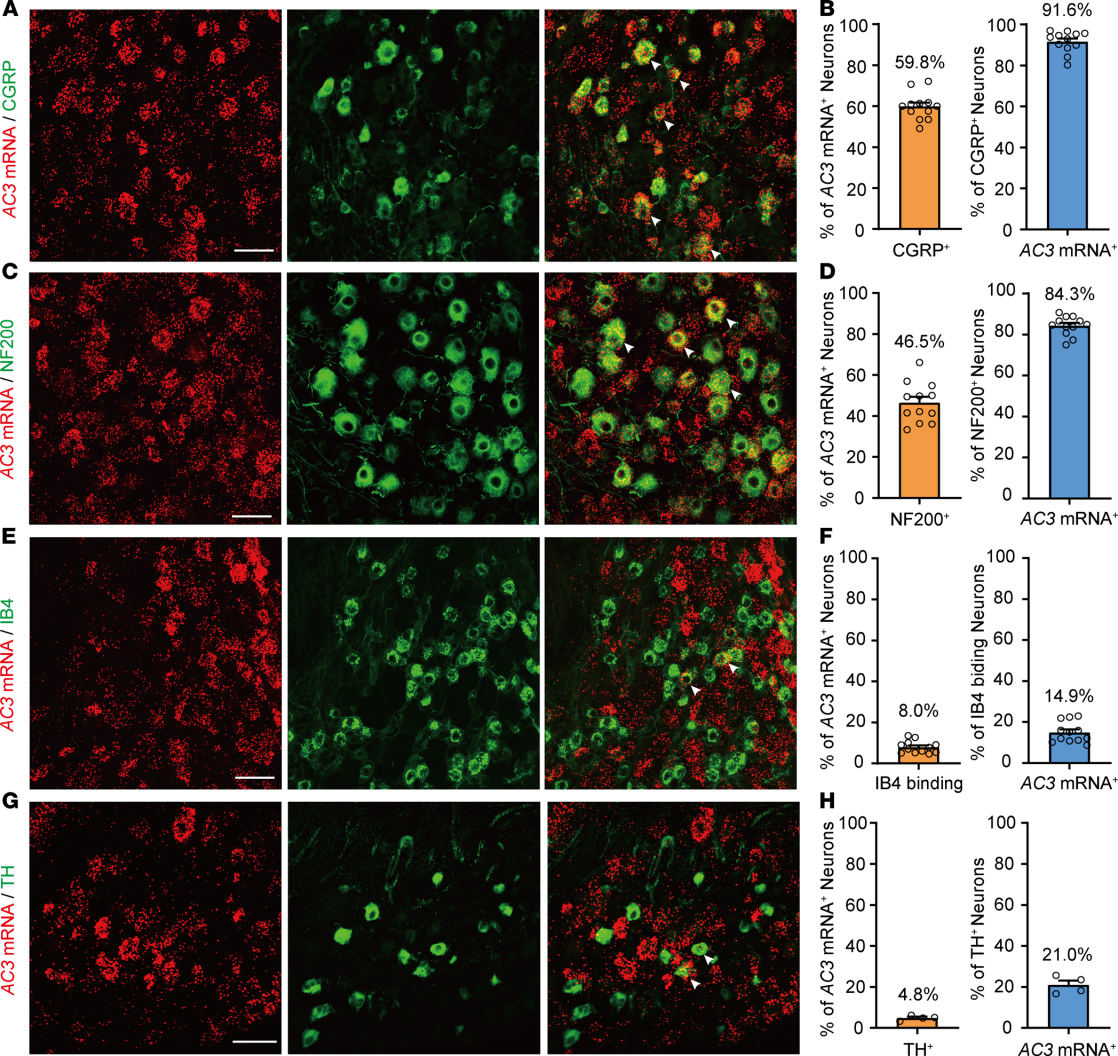
Expression of *AC3* mRNA in the lumbar DRGs in mice. (**A**–**H**) RNAscope in situ hybridization (ISH) combined with IHC showing colocalization and proportion of *AC3* mRNA with CGRP (**A** and **B**), NF200 (**C** and **D**), IB4 (**E** and **F**), and TH (**G** and **H**) immunoreactivities in L3 and L4 DRG neurons. Arrowheads indicate the colocalization of mRNA-positive signals with immunoreactivity-positive signals. Scale bar: 50 μm.

Our previous study showed that AC3 immunoreactivity was mainly expressed in small- and medium-diameter CGRP^+^ peptidergic sensory neurons in the DRG (25), which are thought to be important in nociceptive transmission (30). Therefore, we further examined whether AC3 in the DRG is involved in peripheral nociception in the present study. First, we confirmed the distribution of *AC3* mRNA in the DRG and compared its proportion in different DRG neurons by RNAscope in situ hybridization (ISH) combined with immunofluorescence labeling. As shown in Figure 1, DRG neurons positive for *AC3* mRNA mainly colocalized with CGRP^+^ and NF200^+^ (a marker for large diameter myelinated neurons) neurons. Few *AC3* mRNA colocalized with IB4-immunoreactive (a marker for nonpeptidergic neurons) and tyrosine hydroxylase–immunoreactive (TH-immunoreactive signals) (Figure 1, A–H).

In addition, AC3 immunoreactivity was also detected within the superficial spinal dorsal horn, colocalized with CGRP^+^ primary afferent terminals in laminae I and II_O_. Few IB4^+^ primary afferent terminals in laminae II_i_ and no PKCγ^+^ neurons soma at the boundary of laminae II and III of the spinal cord (31) expressed AC3 immunoreactivity (Supplemental Figure 1, A–C; supplemental material available online with this article; https://doi.org/10.1172/jci.insight.153191DS1). Consistently, AC3 immunoreactivity was colocalized with CGRP^+^ nerve terminals in the glabrous skin of mice planta (Supplemental Figure 1D). Previous studies have identified that AC3 is specifically expressed in the cilia of olfactory epithelium and primary cilia of neurons in the CNS (2, 21, 22). We also detected AC3 immunoreactivity in the primary cilia of neurons in cultured DRG neurons, colocalized with Arl13b (a marker for primary cilia of neurons), indicating that AC3 expressed in both soma and primary cilia of DRG neurons with a prominence in the soma (Supplemental Figure 1E).

**Figure 2:**
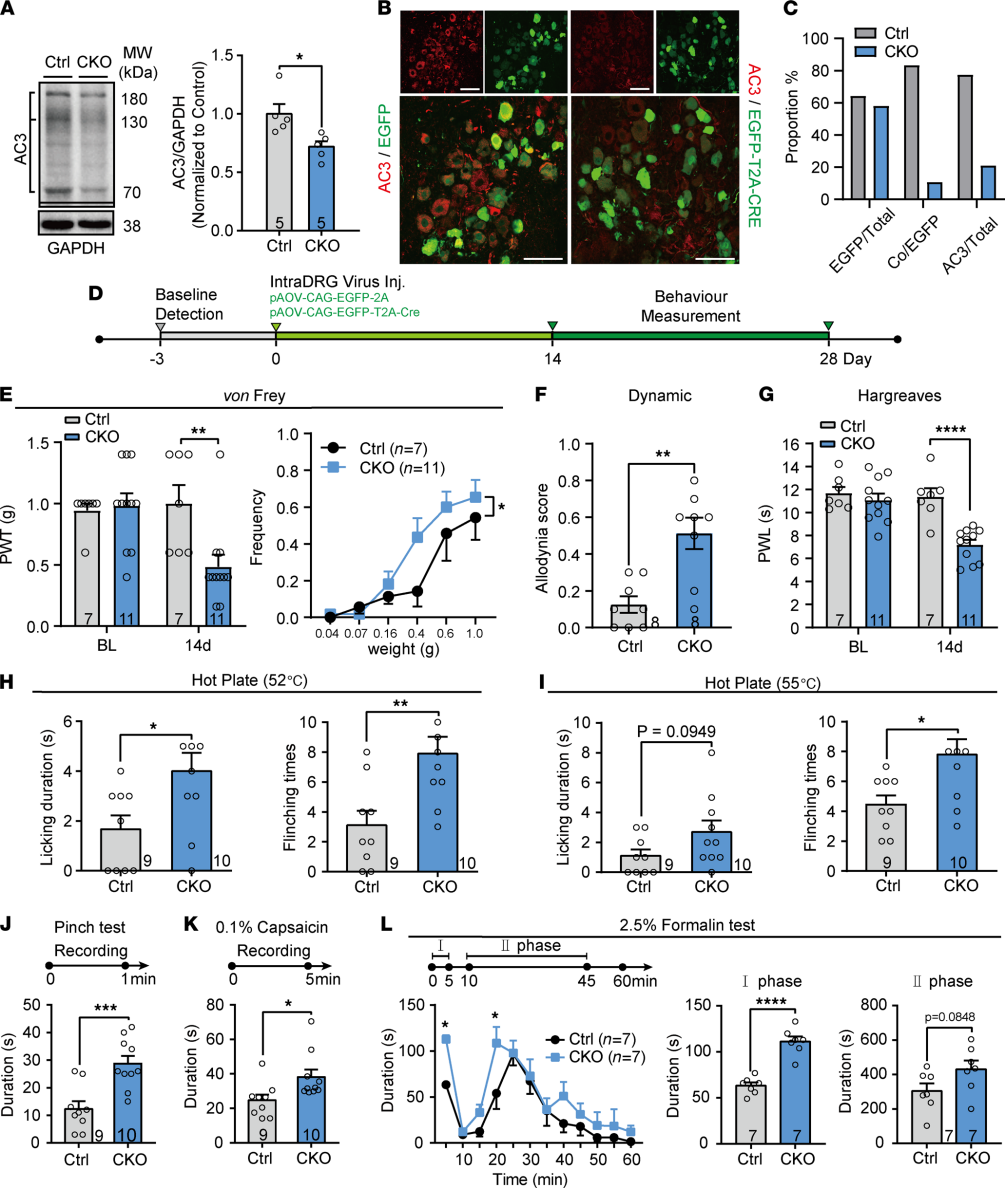
Conditional KO of AC3 in L3 and L4 DRGs facilitates nociceptive behavioral responses in mice. (**A**) Western blot analysis showing a significant decrease in AC3 in L3 and L4 DRGs of CKO mice. The intensity of 3 bands including 180 kDa (glycosylated form), 130 kDa (full-length unglycosylated form), and 70 kDa (monomer form) of AC3 were calculated. *P < 0.05; 2-tailed Student’s *t* test; *n* = 5 Ctrl and CKO (mice). (**B**) AC3-immunoreactivity (AC3-IR, red) colocalized (Co) with EGFP (left) but not with EGFP-T2A-Cre (right) in DRG neurons. Scale bar: 50 μm. (**C**) Quantitative analysis showing proportion of AC3^+^ neurons infected with AAV and AC3 knockdown efficiency. (**D**) Schematic of protocol for virus injection and behavior tests. (**E** and **F**) *AC3* CKO induced mechanical allodynia in von Frey (**E**) and paintbrush (**F**) tests. *P < 0.05, **P < 0.01, 2-tailed Student’s *t* test; *n* = 8 Ctrl and CKO (mice). (**G**) *AC3* CKO induced thermal hyperalgesia. **P < 0.01, ****P < 0.0001; 2-way RM ANOVA followed by Bonferroni’s test; *n* = 7 Ctrl and 11 CKO (mice). (**H**–**K**) *AC3* CKO facilitated nociceptive responses in 52°C (**H**) and 55°C (**I**) hot plate, noxious pinch (**J**) and intraplantar injection (i.pl.) of 0.1% capsaicin (**K**). *P < 0.05, **P < 0.01, ***P < 0.001; 2-tailed Student’s *t* test; *n* = 9 Ctrl and 10 CKO (mice). (**L**) I.pl. 2.5% formalin induced a significantly increased nociceptive response at the I phase in *AC3*-CKO mice. *P < 0.05, ****P < 0.0001; 2-way RM ANOVA followed by Bonferroni’s test (left) and 2-tailed Student’s *t* test (middle and right); *n* = 7 Ctrl and CKO (mice). BL, baseline; Ctrl, Control; CKO, *AC3* CKO.

To address the role of AC3 in peripheral nociception, Cre recombinase expressing adeno-associated virus (pAOV-CAG-EGFP-T2A-Cre) were injected into L3 and L4 DRGs of *AC3^fl/fl^* mice to genetically delete AC3 in L3 and L4 DRG neurons (Supplemental Figure 2, A and B). The control group was injected with AAV expressing EGFP in *AC3^fl/fl^* mice. AC3 protein level and AC3 immunoreactive signals were attenuated in the L3 and L4 DRGs following viral injection (Figure 2, A–C; see complete unedited blots in the supplemental material). Interestingly, *AC3*-CKO mice exhibited a significantly lower paw withdrawal thresholds (PWTs) and a higher response frequency to von Frey hairs on day 14 (Figure 2, D and E; 2-way repeated measures [RM] ANOVA; PWT: *F*_[1,16]_ = 5.16, *P* = 0.04; Frequency: *F*_[1,16]_ = 4.39, *P* = 0.04 for treatment groups). Brush-evoked dynamic mechanical hypersensitivity was also observed in *AC3*-CKO mice (Figure 2F; 2-tailed Student’s *t* test, *t*_[14]_ = 4.01, *P* = 0.0013). Meanwhile, deficiency of AC3 in mouse DRGs also developed thermal hyperalgesia (Figure 2G; 2-way RM ANOVA, treatment groups: *F*_[1,16]_ = 17.62, *P* = 0.0007). Both PWTs to von Frey hairs and paw withdrawal latencies (PWLs) to radiant heat stimulation did not change after intra-DRG injection of control virus in *AC3^fl/fl^* mice (Figure 2, E and G).

Moreover, we compared the responses of peripheral AC3 deletion and control mice to hot plate (52°C and 55°C), pinch plantar, intraplantar (i.pl.) capsaicin (0.1%) and formalin (2.5%) stimuli. In the hot plate test, nociceptive licking duration and flinching times were increased on a 52°C and 55°C plate in *AC3*-CKO mice (Figure 2, H and I). Nociceptive pinch-induced licking, biting, and flinching behaviors were also enhanced in *AC3*-CKO mice (Figure 2J; 2-tailed Student’s *t* test, *t*_[17]_ = 4.11, *P* = 0.0007). In addition, *AC3*-CKO mice showed elevated acute nociceptive responses in capsaicin and formalin tests. The time spent licking, biting, and flinching of the affected paws was obviously prolonged in *AC3*-CKO mice after injection of capsaicin (2-tailed Student’s *t* test, *t*_[17]_ = 2.44, *P* = 0.03) and formalin, especially in the early phase of formalin (Figure 2, K and L; 2-way RM ANOVA; treatment groups: *F*_[1,12]_ = 7.35, *P* = 0.02; time: *F*_[1,12]_ = 18.27, *P* < 0.001 for formalin test response duration; 2-tailed Student’s *t* test, *t*_[12]_ = 7.43, *P* < 0.0001 for I phase response duration in formalin test). All the somatosensory behavioral tests reveal that peripheral AC3–deficient mice developed mechanical, thermal, and inflammatory hyperalgesia.

### Lack of AC3 enhances the excitability of primary sensory neurons.

**Figure 3:**
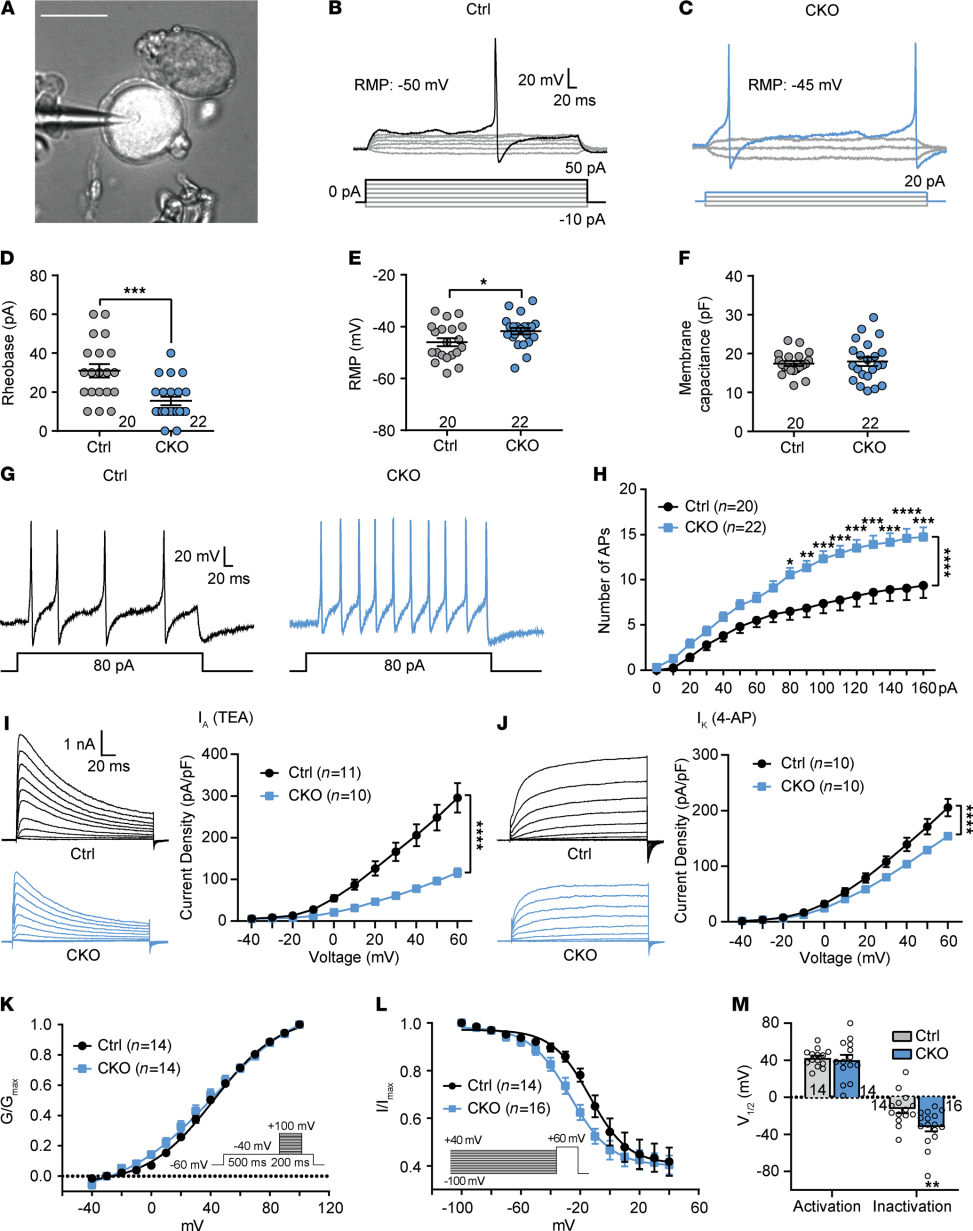
Conditional KO of AC3 enhances the excitability of DRG neurons and decreases the Kv currents. (**A**) Image showing an isolated DRG neuron expressing EGFP with the tip of a pipette during patch clamp recording. Scale bar: 20 μm. (**B** and **C**) Depolarizing current pulse required to evoke an action potential (AP) in control (**B**) and *AC3*-CKO (**C**) DRG neurons. (**D**–**F**) *AC3* CKO reduced the rheobase required to evoke AP (**D**), increased the RMP (more positive; **E**) and had no difference in the membrane capacitance (**F**) of recorded neurons. *P < 0.05, ***P < 0.001; 2-tailed Student’s *t* test; *n* = 20 Ctrl and 22 CKO (cells). (**G**) Examples of the AP traces from control and *AC3*-CKO neurons. (**H**) *AC3* CKO increased the number of APs evoked by current injection. *P < 0.05, **P < 0.01, ***P < 0.001, ****P < 0.0001; 2-way RM ANOVA followed by Bonferroni’s test; *n* = 20 Ctrl and 22 CKO (cells). (**I** and **J**) Pharmacologically separated *I*_A_ (**I**) and *I*_K_ (**J**) in control and *AC3*-CKO DRG neurons under different holding voltage. AC3 deficiency robustly attenuated *I*_A_ (**I**) and *I*_K_ (**J**) densities of DRG neurons. ***P < 0.001; 2-way RM ANOVA followed by Bonferroni’s test; *n* = 8–11 (cells). (**K**–**M**) *AC3* CKO did not shift the steady-state activation curve and half-activation voltage of Kv channels (**K** and **M**), but it left-shifted the steady-state inactivation curve of Kv channels toward hyperpolarizing direction and decreased half-inactivation voltage (more negative) in DRG neurons (**L** and **M**). **P < 0.01; 2-tailed Student’s *t* test; *n* = 14–16 (cells).

To address whether AC3 deficiency would alter the excitability of DRG neurons, we performed whole-cell patch clamp recordings in *AC3* CKO and control (EGFP) DRG small-diameter (<25 μm) neurons (Figure 3A). Action potentials (APs) were evoked by superimposed positive current steps. A lower rheobase (2-tailed Student’s *t* test, *t*_[40]_ = 3.88, *P* = 0.0004) and a leftward shift in the input-output curve of APs (2-way ANOVA, *F*_[1,40]_ = 141, *P* < 0.0001) with a more positive resting membrane potential (RMP) (2-tailed Student’s *t* test, *t*_[40]_ = 2.11, *P* = 0.04) were observed in AC3-deleted neurons (Figure 3, B–H). No significant differences in the membrane capacitance were detected between *AC3* CKO and control neurons (Figure 3F). We also recorded EGFP^–^ DRG neurons from Cre- and control-virus injected mice. No differences in RMP, membrane capacitance, and the threshold and frequency of APs were found, suggesting that the excitability of AC3^–^ or innocent AC3^+^ neurons remained intact both in Cre- or control virus–injected mice (Supplemental Figure 3).

Kv channels are crucial determinants of excitability of DRG neurons (32). Consistent with our previous study in rats (33), 2 primary subpopulations of Kv currents were identified in acute isolated mouse DRG neurons based on different kinetic properties. These 2 subpopulations of Kv currents — including rapidly inactivating A-type potassium currents sensitive to 4-AP (*I*_A_) and B-type sustained delayed potassium currents sensitive to TEA (*I*_K_) — were confirmed through pharmacological method (Supplemental Figure 4, A–E). DRG neurons dominated by A- and B-type Kv currents accounted for about 56.45% (35 of 62) and 43.55% (27 of 62), respectively, of all the recorded small-diameter DRG neurons (Supplemental Figure 4B). Interestingly, B-type Kv current–dominated DRG neurons had significantly larger membrane capacity than A-type potassium current–dominated DRG neurons, which suggests that DRG neurons dominated by 2 types of Kv currents are 2 different populations with different cell diameters (Supplemental Figure 4C). Whole-cell patch clamp recordings showed that deletion of AC3 robustly decreased *I*_A_ and *I*_K_ current densities in *AC3* CKO DRG neurons (Figure 3, I and J; 2-way ANOVA; *F*_[1,19]_ = 127.1, *P* < 0.0001 for *I*_A_; *F*_[1,19]_ = 42.02, *P* < 0.0001 for *I*_K_). Western blot analysis showed that Kv subtypes Kv1.4, Kv3.4, and Kv4.3 were all decreased in the DRG of *AC3*-CKO mice (Supplemental Figure 4, F–K; see complete unedited blots in the supplemental material). For further understanding the effect of AC3 deletion on gating kinetics of Kv channels in DRG neurons, we analyzed the steady-state activation and inactivation properties of the channels. Cells were held at –80 mV and depolarized in 10 mV steps from –40 mV to 100 mV at 10-second intervals (Figure 3K). The peak current evoked from each voltage step was transformed into corresponding conductance, and the Kv activation curve was obtained by normalizing each conductance to the maximal conductance (G/G_max_) fitted with Boltzmann function. No shift was found in the voltage-dependent activation curve in AC3-deleted neurons compared with controls (Figure 3K). Steady-state inactivation of the Kv channel was determined using 1-second conditioning prepulse from –100 mV to 40 mV membrane potentials followed by a 200-ms test pulse of 60 mV in DRG neurons (Figure 3L). The inactivation curve was obtained with each peak current normalized to the maximal peak current. AC3 deletion caused a leftward shift toward the hyperpolarizing potential of the inactivation curve (Figure 3L). The half-maximal (voltage [V]_1/2_) activation potentials were similar between *AC3* CKO and control groups, whereas V_1/2_ inactivation potentials were more hyperpolarized in AC3-deleted neurons (Figure 3M; 2-tailed Student’s *t* test, *t*_[28]_ = 2.872, *P* = 0.0077).

Immunofluorescence also showed that about 51.1% of AC3^+^ DRG neurons colocalized with TRPV1 (Supplemental Figure 5, A and B). TRPV1 has been demonstrated to play a crucial role in peripheral sensitization (34, 35). Whole-cell patch clamp recordings showed that capsaicin-induced (1 μM) TRPV1 currents in small-diameter (<25 μm) *AC3*-CKO DRG neurons significantly increased (Supplemental Figure 5, C and D). Successive applications of 1 μM capsaicin (3 seconds, with interval of 60 seconds) produced a progressive decline or desensitization in the responses in the neurons from both control and *AC3*-CKO mice. The desensitization rate had no significant difference between *AC3*-CKO DRG neurons and controls (Supplemental Figure 5, C and E). These data imply that the neuronal excitability and sensitivity to capsaicin of DRG neurons were increased after deletion of AC3, consistent with the behavioral hypersensitivity.

**Figure 4:**
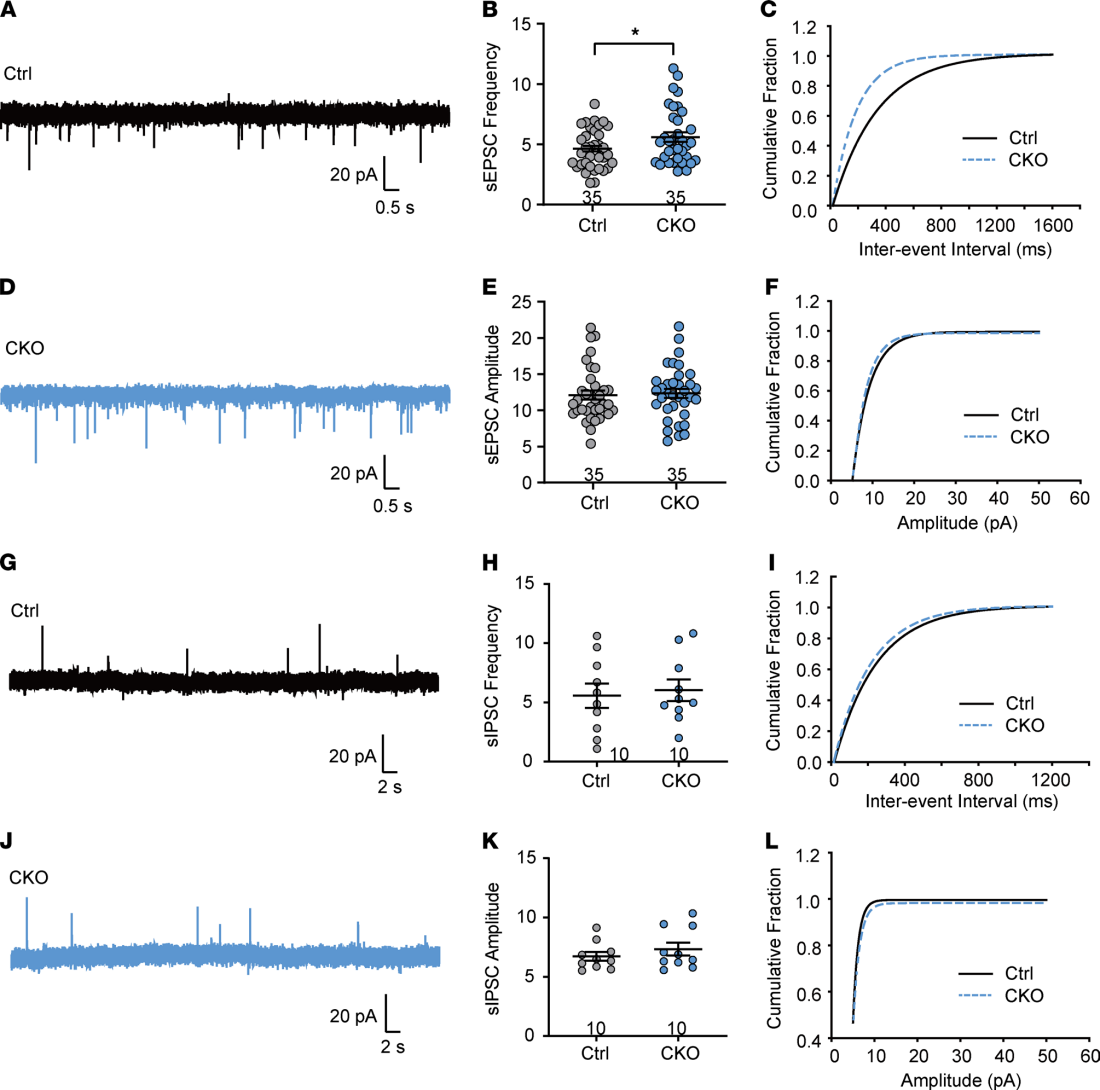
*AC3* CKO alters excitatory synaptic transmission in spinal cord slices. (**A**–**C**) Patch clamp recording of sEPSC showing an increase in the frequency of sEPSCs in spinal lamina II_o_ neurons (**A** and **B**) and a left-shifted the cumulative fraction of interevent interval (**C**) *AC3*-CKO mice. *P < 0.05; 2-tailed Student’s *t* test; *n* = 35 Ctrl and 35 CKO (cells). (**D**–**F**) *AC3* CKO did not affect the amplitude (**D** and **E**) and corresponding cumulative fraction (**D** and **F**) of sEPSCs. (**G**–**L**) Patch clamp recording of sIPSC showing no difference in the frequency (**G**–**I**) and amplitude (**J**–**L**) of sIPSCs in spinal lamina II_o_ neurons between control and *AC3*-CKO mice. 2-tailed Student’s *t* test; *n* = 10 Ctrl and 10 CKO (cells).

As described above, AC3 was also expressed in the primary afferent terminals of superficial spinal dorsal horn (Supplemental Figure 1, A–C), suggesting the involvement of AC3 in the regulation of spinal synaptic transmission. We recorded spontaneous excitatory postsynaptic currents (sEPSCs) and spontaneous inhibitory postsynaptic currents (sIPSCs) in lamina II_o_ neurons in spinal cord slices from control and *AC3*-CKO mice. As compared with controls, AC3 deletion induced an increased frequency of sEPSCs (2-tailed Student’s *t* test, *t*_[68]_ = 2.12, *P* = 0.04), but not amplitude of sEPSCs, suggesting that ablation of AC3 in DRG neurons increased presynaptic glutamate release in the spinal dorsal horn (Figure 4, A–F). No significant difference was found in frequency and amplitude of sIPSCs between control and *AC3*-CKO mice (Figure 4, G–L).

### Ablation of AC3 upregulates AC1 expression and function in the DRG.

**Figure 5:**
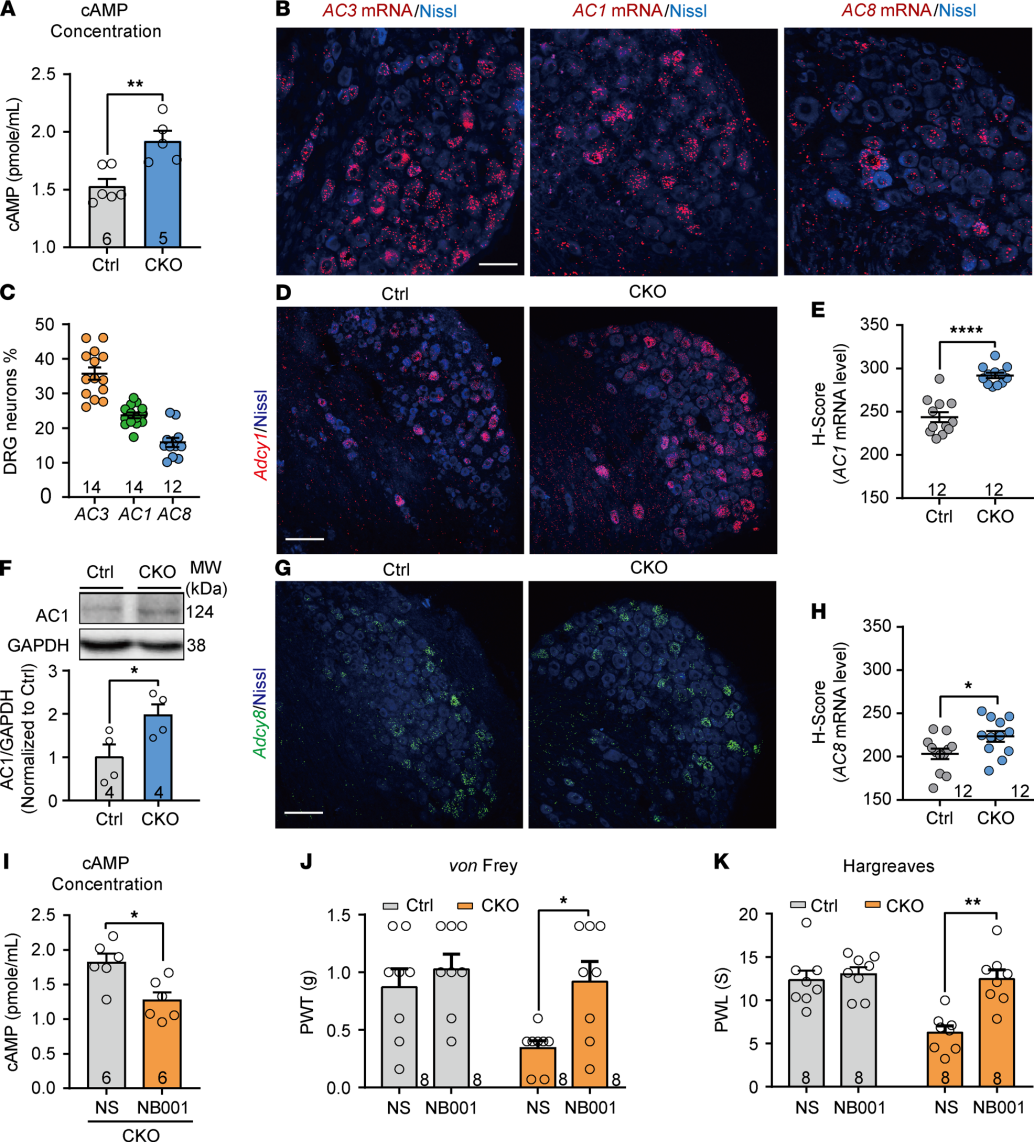
The levels of cAMP concentration and expression of AC1 and AC8 in the DRG. (**A**) cAMP concentration was markedly increased in *AC3*-CKO DRGs. **P < 0.01; 2-tailed Student’s *t* test; *n* = 6 Ctrl and 5 CKO (mice). (**B** and **C**) RNAscope ISH showing the expression levels of *AC3*, *AC1*, and *AC8* mRNA in DRG neurons. Scale bar: 50 μm. (**D** and **E**) RNAscope ISH showing a robustly increased expression level of *AC1* mRNA in *AC3*-CKO DRGs. ****P < 0.0001; 2-tailed Student’s *t* test; *n* = 12 Ctrl and 12 CKO (DRG slices from 4 mice). (**F**) Western blot analysis showing the increased AC1 protein level in *AC3*-CKO DRGs. Data are represented as fold changes compared with the intensity of GAPDH. *P < 0.05; 2-tailed Student’s *t* test; *n* = 4 Ctrl and 4 CKO (mice). (**G** and **H**) RNAscope ISH showing an increased expression level of *AC8* mRNA in *AC3*-CKO DRGs. *P < 0.05; 2-tailed Student’s *t* test; *n* = 12 Ctrl and 12 CKO (DRG slices from 4 mice). (**I**) Lumbar puncture of AC1 antagonist NB001 (2.5 μg) significantly blocked *AC3*-CKO–induced upregulation of cAMP concentration in the DRGs. *P < 0.05; 2-tailed Student’s *t* test; *n* = 6 Ctrl and 6 CKO (mice). (**J** and **K**) NB001 (2.5 μg) significantly reversed *AC3*-CKO–induced the mechanical allodynia (**J**) and thermal hyperalgesia (**K**). *P < 0.05, **P < 0.01; 2-way RM ANOVA followed by Bonferroni’s test; *n* = 8 Ctrl and 8 CKO (mice).

**Figure 6:**
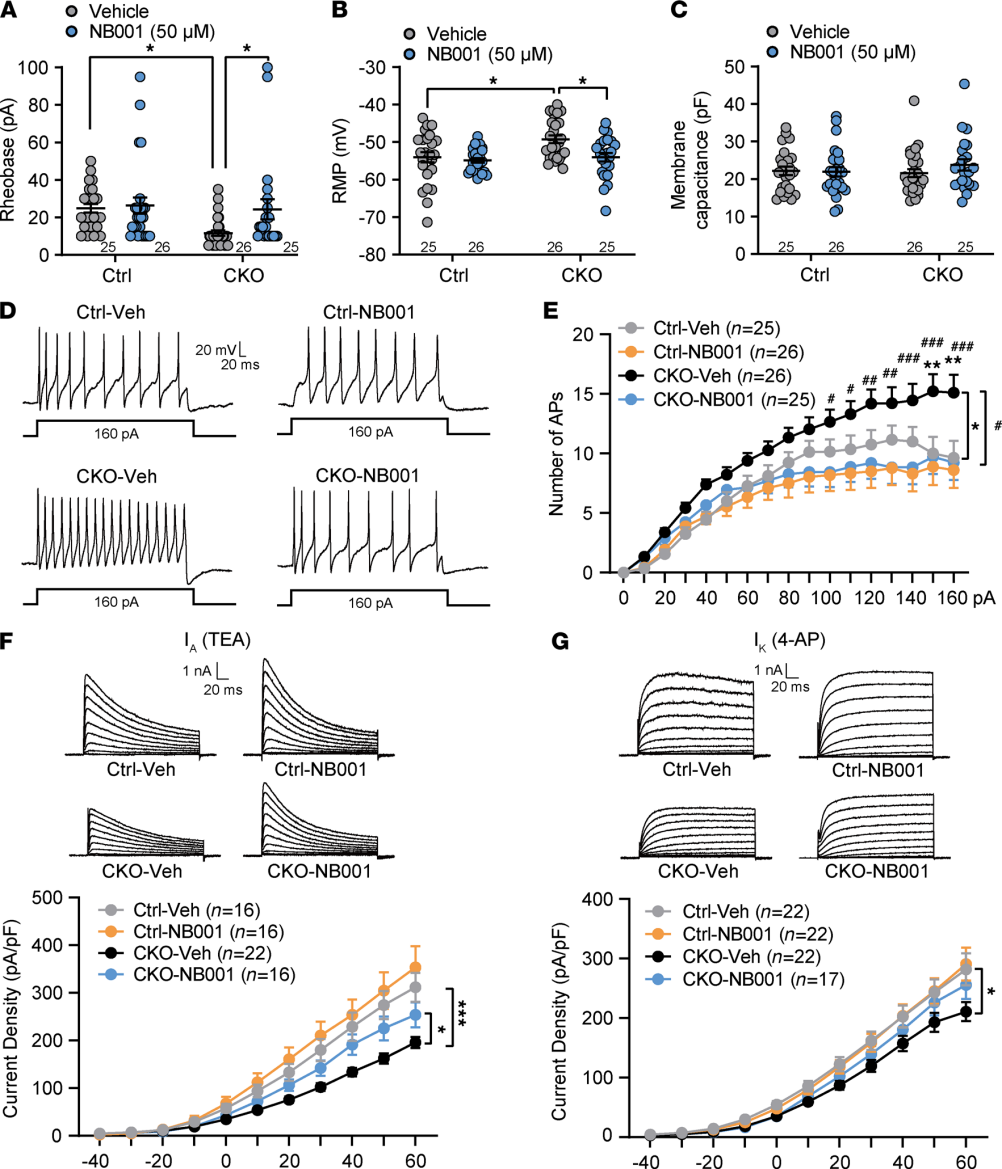
Inhibition of AC1 reversed the enhanced neuronal excitability and decreased Kv currents in AC3-deleted DRG neurons. (**A**–**C**) Bath application of AC1 specific inhibitor NB001 (50 μM) for 30 minutes. *AC3*-CKO–induced attenuation of rheobase required to evoke AP (**A**) and RMP (more hyperpolarization (**B**) were reversed in DRG neurons. The membrane capacitance had no difference of recorded neurons (**C**). *P < 0.05; 1-way ANOVA followed by post hoc Dunnett’s multiple-comparison test; *n* = 25 (Ctrl-Veh, CKO-NB001) and 26 (Ctrl-NB001, CKO-Veh, cells). (**D**) Examples of AP traces from control and *AC3*-CKO DRG neurons with vehicle (Veh) or NB001 application. (**E**) NB001 reduced the increased number of APs evoked by current injection in AC3 deleted DRG neurons. *P < 0.05, **P < 0.01, CKO-Veh versus Ctrl-Veh; ^#^*P* < 0.05, ^##^*P* < 0.01, ^###^*P* < 0.001, CKO-NB001 versus CKO-Veh; 2-way RM ANOVA followed by Bonferroni’s test; *n* = 25 (Ctrl-Veh, CKO-NB001) and 26 (Ctrl-NB001, CKO-Veh, cells). (**F** and **G**) NB001 significantly increased *I*_A_ (**F**) and slightly increased *I*_K_ (**G**) densities in AC3-deleted DRG neurons. *P < 0.05, ***P < 0.001; 2-way RM ANOVA followed by Bonferroni’s test; *n* = 16 (Ctrl-Veh, Ctrl-NB001, CKO-NB001) and 22 (CKO-Veh, cells).

The activation of AC leads to an increase in cAMP, which acts as an intracellular second messenger playing a crucial role in various physiological and pathological functions. Increased cAMP level is known to be associated with nociception, and agents that decreased cAMP have analgesic effects (36). However, the present study showed an unconventional result that deficiency of AC3 in DRG neurons resulted in nociceptive facilitation. We, therefore, measured cAMP concentration in DRGs from *AC3*-CKO and control mice. Unexpectedly, DRG cAMP concentration was significantly increased following AC3 deletion in DRG neurons (Figure 5A; 2-tailed Student’s *t* test, *t*_[68]_ = 3.81, *P* = 0.004). This result suggests that other subtypes of the AC family (e.g., AC1 and AC8) may be enhanced at a compensable rate following AC3 deletion. Studies have shown that depletion of *AC1* or *AC1/AC8* DKO, but not *AC8* KO, attenuated inflammation and chronic muscle pain (14, 19), and all 3 Ca^2+^/calmodulin-stimulated ACs mRNA were detected in the rat DRG (37, 38). We compared the expression levels of *AC1*, *AC3*, and *AC8* mRNA in the DRG by RNAscope ISH. The mRNA of all 3 ACs was detected in mouse DRG neurons, with the highest proportion of *AC3* mRNA among the 3 (Figure 5, B and C). Interestingly, after deficiency of AC3, *AC1* mRNA (2-tailed Student’s *t* test, *t*_[22]_ = 7.23, *P* < 0.0001) and protein levels (2-tailed Student’s *t* test, *t*_[6]_ = 2.49, *P* = 0.047) were significantly upregulated in DRG neurons (Figure 5, D–F; see complete unedited blots in the supplemental material.). AC8 mRNA was also partially upregulated in the DRG after AC3 deletion (Figure 5, G and H; 2-tailed Student’s *t* test, *t*_[22]_ = 2.34, *P* = 0.03). To confirm whether AC3 deletion–induced cAMP upregulation is caused by elevated AC1, specific AC1 inhibitor NB001 (2.5 μg) was injected into *AC3*-CKO mice by lumbar puncture. As shown in Figure 5I, NB001 significantly reduced cAMP concentration (2-tailed Student’s *t* test, *t*_[10]_ = 3.16, *P* = 0.01). Consistently, NB001 also rescued the mechanical allodynia and thermal hyperalgesia induced by AC3 ablation (Figure 5, J and K; 2-way RM ANOVA; PWT: *F*_[1,14]_ = 8.18, *P* = 0.01; PWL: *F*_[1,14]_ = 13.49, *P* = 0.003). Whole-cell patch clamp recording further showed that bath application NB001 (50 μM) enhanced the rheobase (1-way ANOVA, *F*_[3,98]_ = 3.927, *P* = 0.0108) and decreased AP firing frequency (2-way ANOVA, *F*_[1,49]_ = 6.680, *P* = 0.0128) with a more negative RMP (1-way ANOVA, *F*_[3,98]_ = 5.952, *P* = 0.0009) in AC3-deleted DRG neurons (Figure 6, A–E). These results indicate that enhanced excitability of AC3 deleted DRG neurons could be reversed by inhibition of AC1. Moreover, NB001 significantly increased *I*_A_ current densities in AC3-deleted DRG neurons (Figure 6F; 2-way ANOVA, *F*_[1,36]_ = 5.860, *P* = 0.0207). The decreased *I*_k_ current densities by *AC3* CKO were also partially reversed by NB001, although it was not statistically significant (Figure 6G). These findings suggest that elevated cAMP, decreased Kv channel currents, enhanced neuronal excitability, and nociceptive hypersensitivity by AC3 deficiency may be mediated by compensatory enhancement of AC1.

### Peripheral AC3 is involved in the κ-opioid receptor–mediated analgesia.

AC is an effector of GPCRs. Opioid analgesia is mediated by opioid receptors, such as MOR (μ opioid receptor), DOR (δ opioid receptor), and KOR (κ opioid receptor), which belong to the family of GPCRs. To address whether AC3 is associated with opioid-mediated antinociception, we examined the analgesic effects of MOR agonist DAMGO, DOR agonist [D-Ala2]-deltorphin II (39), and KOR agonist nalfurafine hydrochloride (40) in *AC3*-CKO and control mice.

**Figure 7:**
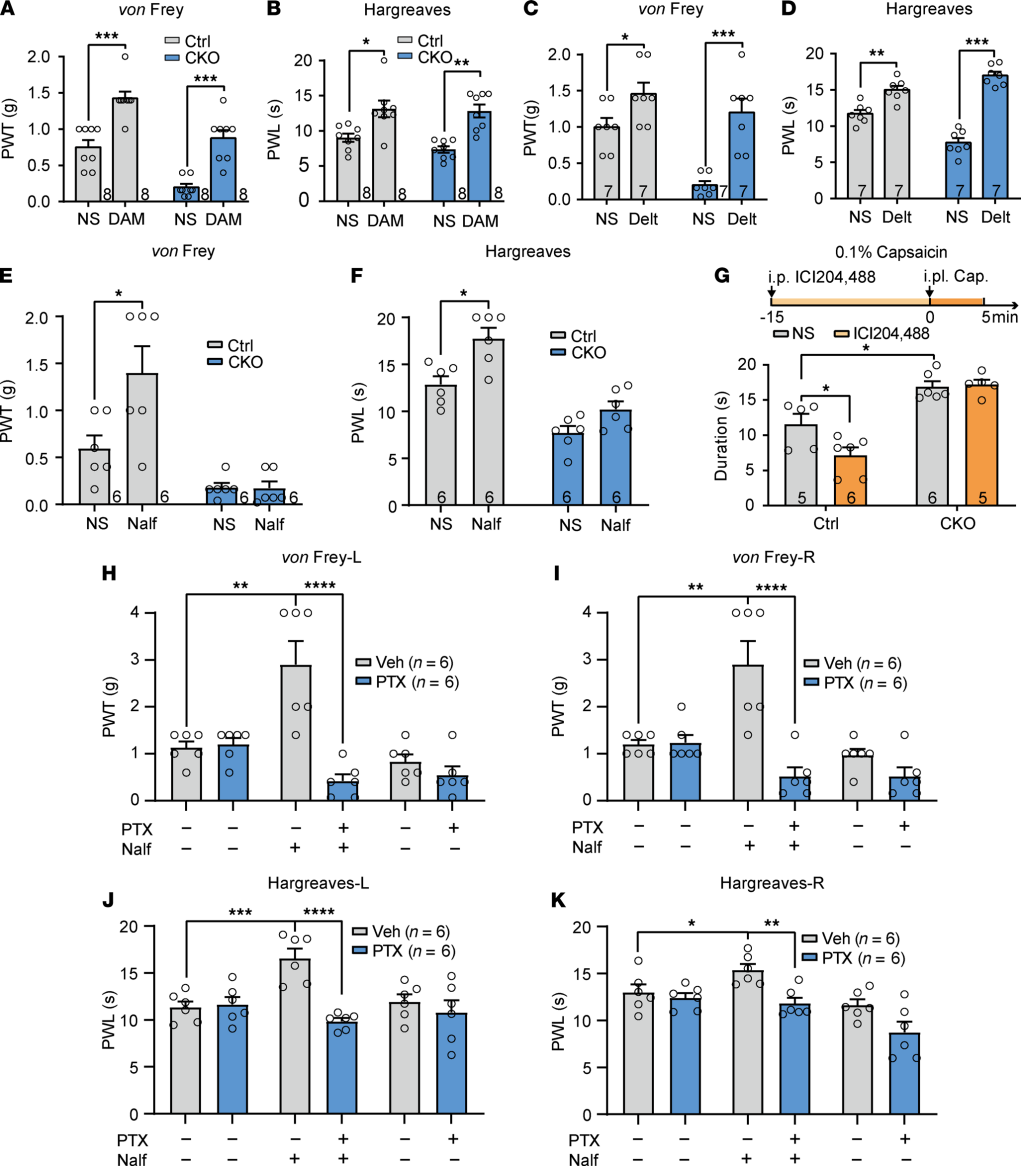
AC3 mediates KOR-induced analgesia peripherally in mice. (**A**–**D**) Lumbar puncture of MOR agonist DAMGO (15 ng) (**A** and **B**) and DOR agonist [D-Ala2]-deltorphin II (5 μg) (**C** and **D**) elevated paw withdrawal thresholds (PWTs) (**A** and **C**) and paw withdrawal latencies (PWLs) (**B** and **D**) in both control and *AC3*-CKO mice. *P < 0.05, **P < 0.01, ***P < 0.001; 2-way RM ANOVA followed by Bonferroni’s test; *n* = 7 or 8 Ctrl and CKO (mice). (**E** and **F**) Lumbar puncture KOR agonist nalfurafine (0.5 μg) significantly enhanced PWTs (**E**) and PWLs (**F**) in control mice but had no effect in *AC3*-CKO mice. *P < 0.05; 2-way RM ANOVA followed by Bonferroni’s test; *n* = 6 Ctrl and 6 CKO (mice). (**G**) I.p. injection of KOR agonist ICI204,488 (10 mg/kg) significantly attenuated capsaicin-induced nociceptive responses but had no effect in *AC3*-CKO mice. *P < 0.05; 2-way RM ANOVA followed by Bonferroni’s test; *n* = 5 or 6 Ctrl and CKO (mice). (**H**–**K**) Lumbar puncture of PTX (0.1 μg) blocked KOR agonist–induced increases in PWTs (**H** and **I**) and PWLs (**J** and **K**). *P < 0.05, **P < 0.01, ***P < 0.001, ****P < 0.0001; 2-way RM ANOVA followed by Bonferroni’s test; *n* = 6 Veh and 6 PTX 0.1 μg (mice). Veh, vehicle; L, left paw; R, right paw; DAM, DAMGO; Delt, [D-Ala2]-deltorphin II; Nalf, nalfurafine; NS, normal saline.

Lumbar puncture application (i.t.) of DAMGO (15 ng) and [D-Ala2]-deltorphin II (5 μg) significantly reversed AC3 deficiency–induced mechanical allodynia and thermal hyperalgesia in *AC3*-CKO mice, and it also elevated PWTs and PWLs in control mice, suggesting that AC3 deletion did not affect MOR- and DOR-mediated antinociception (Figure 7, A–D). Conversely, KOR agonist nalfurafine (0.5 μg) significantly increased PWLs and PWTs in control mice but had no effect in *AC3*-CKO mice (Figure 7, E and F). Although it has been reported that drugs can be predominantly delivered into DRG neurons by direct lumbar puncture (41), we also chose peripherally restricted KOR agonist ICI204,488 in order to further confirm the role of AC3 in peripheral KOR analgesia. Intraperitoneal (i.p.) injection of ICI204,488 (10 mg/kg) significantly reduced i.pl. capsaicin-induced licking, biting, and flinching behaviors in control mice, whereas these pain-like behaviors had no difference between vehicle and ICI204,488 treatment in *AC3*-CKO mice (Figure 7G). These findings indicate that DRG AC3 contributes to KOR-mediated analgesic effect peripherally. Moreover, lumbar puncture of pertussis toxin (PTX; 0.1 μg), an irreversible inhibitor of Gα_i/o_ protein, completely blocked KOR agonist–induced (nalfurafine-induced) antinociceptive effects (Figure 7, H–K), suggesting that KOR agonist may suppress nociception through Gα_i/o_ protein inhibition of AC3 activity.

### AC3 mediates KOR agonist–induced enhancement of Kv channel currents in DRG neurons.

A prominent mechanism underlying peripheral opioid analgesia is the activation of potassium channels in DRG neurons (42, 43). Therefore, we examined the effect of KOR agonists on Kv channels in control and *AC3*-CKO DRG neurons.

**Figure 8:**
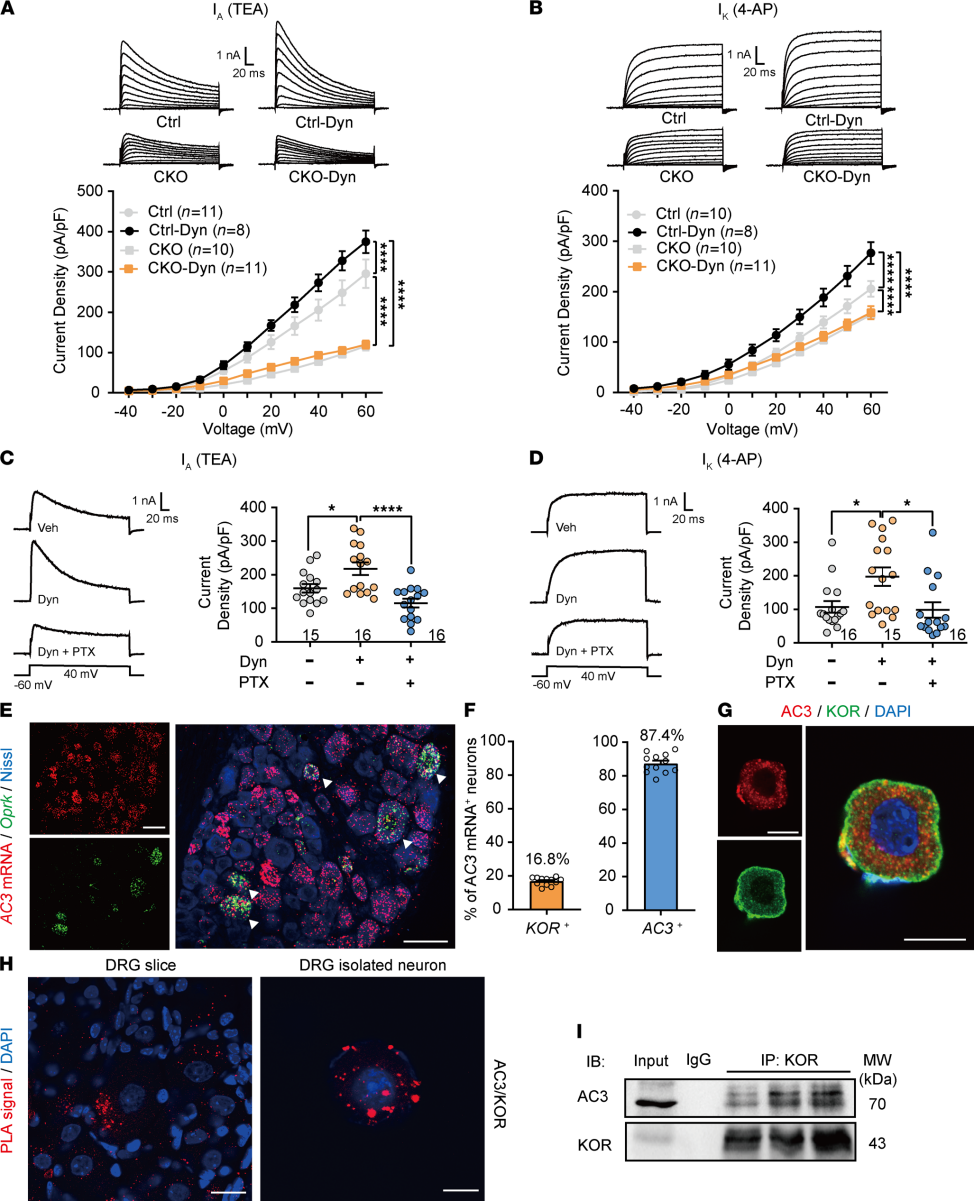
AC3 mediates KOR-induced enhancement of Kv channel currents in DRG neurons. (**A** and **B**) Dynorphin A significantly increased *I*_A_ (**A**) and *I*_K_ (**B**) densities in control DRG neurons but not in *AC3*-CKO DRG neurons. ****P < 0.0001; 2-way RM ANOVA followed by Bonferroni’s test; *n* = 8–11 (cells). (**C** and **D**) PTX (Gα_i/o_ protein irreversible inhibitor, 1 mM) in the intracellular solution completely abolished the dynorphin A–induced increases in *I*_A_ (**C**) and *I*_K_ (**D**) in naive DRG neurons. *P < 0.05, ****P < 0.0001; 1-way ANOVA; *n* = 15–16 (cells). Dyn, dynorphin A. (**E** and **F**) RNAscope ISH showing colocalization and proportion of DRG neurons positive for *AC3* mRNA (*Adcy3*) with *KOR* mRNA (*Oprk*). Scale bar: 50 μm. Arrowheads indicate the colocalization of positive signals for AC3 mRNA and KOR mRNA. (**G**) Double immunofluorescence of isolated DRG neurons showing the colocalization of AC3-IR with KOR-IR. Scale bar: 10 μm. (**H**) Proximity ligation assay (PLA) showing close interaction signals of AC3 with KOR in DRG slice (left) and isolated DRG neurons (right). Scale bar: 20 μm (left) and 10 μm (right). (**I**) Co-IP displaying that AC3 (IB) and KOR (IB) are captured by mouse anti-KOR antibody (IP) in mouse lumbar DRGs. Normal mouse IgG IP was applied as the negative control. *n* = 3 (mice).

Whole-cell patch clamp recordings showed that endogenous KOR agonist dynorphin A (Dyn, 1 μM) significantly increased *I*_A_ and *I*_K_ current densities in DRG neurons from control mice (Figure 8, A and B; 2-way ANOVA; *I*_A_: *F*_[3,36]_ = 152.2, *P* < 0.0001; *I*_K_: *F*_[3,35]_ = 75.8, *P* < 0.0001 for treatment groups). However, neither *I*_A_ nor *I*_K_ current densities could be altered by Dyn in DRG neurons lacking AC3, suggesting that AC3 is necessary for KOR-induced amplification of Kv currents (Figure 8, A and B). Furthermore, Dyn–induced increases in *I*_A_ and *I*_K_ current densities were prevented by PTX (1 mM), an irreversible inhibitor of Gα_i/o_ protein, indicating that KOR agonist increased Kv currents via Gα_i/o_ protein (Figure 8, C and D).

Furthermore, we detected a colocalization of AC3 and KOR in DRG neurons. RNAscope strategy showed that about 87.4% of DRG neurons positive for *KOR* mRNA were colocalized with *AC3* mRNA (Figure 8, E and F). Immunofluorescence staining confirmed the colocalization of AC3 and KOR in both membrane and cytoplasm of isolated DRG neurons (Figure 8G). Most notably, we identified the interaction relationship between AC3 and KOR in DRG neurons by proximity ligation assay (PLA) strategy and co-IP experiments. PLA is a relatively sensitive technique that detects colocalization of proteins in tissues or cells. Many PLA signals were observed through interaction of AC3 and KOR antibodies combined PLA detection probes in DRG slices and isolated DRG neurons (Figure 8H). Conversely, only sparse PLA signals were detected through interaction of AC1 and KOR in isolated DRG neurons (Supplemental Figure 6A). Co-IP shows that monomer of membrane AC3 (about 70 kDa) (Figure 8I) but not AC1 (Supplemental Figure 6B) and KOR was captured by anti-KOR antibody in DRG homogenate. In addition, we use Discovery Studio to conduct homology modeling of AC3 sequences. The ZDOCK module of Discovery Studio was used to perform protein-protein docking with KOR. The predicted docking results show that the 170Arg-196Leu α-helical structure on KOR combined with AC3 940ILE-952LEU β-turn structure and the 1123Asp-1142asp α-helical structure (Supplemental Figure 6, C and D). These data suggest that, in addition to the classical Gα_i/o_ signaling pathway, KOR agonists may also inhibit the AC3 directly through protein-protein interaction.

## Discussion

AC-cAMP signaling pathways in GPCR-mediated regulation have been shown to play a crucial role in nociceptive processing (36). Many isoforms of ACs have been identified in the CNS (1). Although ubiquitously expressed ACs often are excluded as drug targets due to concerns of their side effects, different distribution of AC isoforms in specific population of neurons in the CNS makes them gather more attention as potential regulators of selective GPCR signaling pathways (28). In this study, we report an unconventional role of peripheral AC3 in regulation of neural excitability and nociceptive behaviors in mice. We further reveal that KOR-mediated analgesia is achieved by AC3 regulating Kv channels in DRG neurons.

### AC3 CKO increases the excitability and decreases Kv channel currents of DRG neurons, resulting in hyperalgesia.

Previous studies have shown that AC3 was restrictedly expressed in the olfactory sensory cilia and primary cilia of neurons throughout the CNS (4, 22). A recent study from our laboratory showed that conditional KO of AC3 in somatostatin^+^ (SST^+^), but not parvalbumin^+^ (PV^+^) interneurons induced depressive-like behaviors in mice (24). Intriguingly, our previous (25) and current studies showed that DRG neurons also expressed a large amount of AC3, especially in the soma of DRG neurons. What’s more, *AC3* mRNA expression was much more than that of *AC1* and *AC8* in the lumbar DRGs, suggesting that AC3 plays an important role in peripheral sensory afferent. In the DRGs, *AC3* mRNA was predominantly expressed in CGRP^+^ small- to medium-diameter peptidergic neurons. Genetic ablation of CGRPα-expressing sensory neurons has been reported to reduce sensitivity to noxious heat and capsaicin (44). Single-cell RNA transcriptome analyses combined with functional tests further suggest that CGRP^+^ DRG neurons act as polymodal nociceptors (45–47). We conditionally knocked out AC3 in L3 and L4 DRGs by employing a cre-loxp system, which produced a significant nociceptive hypersensitivity in multiple behavioral tests, including pinch mechanical stimulation, Hargreaves’ and hot plate thermal stimulation, and capsaicin and formalin chemical stimulation. Corresponding to behavioral responses, ablation of AC3 in small- to medium-diameter DRG neurons significantly increased neuronal excitability, manifesting by increased AP firing frequency, decreased rheobase, and a more positive resting membrane potential. Given that CGPR^+^ terminals in the superficial dorsal horn also expressed strong AC3^+^ signals, we recorded sEPSCs and sIPSCs in lamina II_o_ neurons. The significant increased frequencies of sEPSCs were observed in *AC3*-CKO mice, suggesting that lack of AC3 in DRG neurons led to excitatory synaptic transmission potentiation in the spinal dorsal horn.

Kv channels are crucial for controlling neuronal excitability and nociceptive behaviors (48, 49). Downregulation of Kv currents, including *I*_A_ and *I*_K_ in DRG neurons, caused robust increases in neuronal excitability and responsiveness to nociceptive stimulation and contribute to mechanical and thermal hyperalgesia (32, 50, 51). Consistently, we found that Kv currents were markedly attenuated in *AC3*-CKO DRG neurons. At certain membrane potentials, the available Kv channels were decreased and the expression of Kv subtypes was downregulated after KO of AC3 (Supplemental Figure 7).

It is worth noticing that *AC3* mRNA was also colocalized with NF200. Despite approximately 28% of CGRP^+^ neurons expressed NF200 (47), NF200 is generally expressed in the medium- to large-sized nonnociceptors with myelinated, fast-conducting A-fibers in low-threshold mechanosensation (LTMRs) (52), indicating that AC3 is involved in mechanical allodynia. In support, our current study shows that KO of AC3 in L3 and L4 DRGs results in a robust mechanical allodynia with a lower PWT and a higher response frequency tested by von Frey hairs and a higher dynamic mechanical response score in paintbrush test.

### AC3 CKO increases cAMP in the DRGs.

cAMP converted from ATP is catalyzed by ACs and, as the first discovered cellular second messenger, was implicated in nociceptor sensitization (36). Intradermal injection of membrane-permeable cAMP analogs (53) or the AC activator forskolin (54) produces sensitization of nociceptive fibers resulting in hyperalgesia (55). Consistent with the positive correlation between the hyperalgesia and increased cAMP level, the present study showed a significantly upregulation of cAMP concentration and nociceptive behavioral hypersensitivity after deletion of AC3 in the DRGs. Despite that all the AC1, AC3, and AC8 are Ca^2+^/calmodulin-stimulated Group I ACs, AC3 is a Ca^2+^-inhibited AC in vivo, unlike AC1 and AC8, which are Ca^2+^-activated ACs (56). Submicromolar concentrations of intracellular free Ca^2+^ inhibited receptor-stimulated AC3 by Ca^2+^-stimulated kinase (57). When noxious stimuli activates sensory neurons in the DRG, increased intracellular Ca^2+^ may transiently inhibit AC3 and the subsequent cAMP. This may partly explain the increase in cAMP after ablation of AC3 in the DRGs.

Another important finding of the current study is that *AC1* mRNA and protein were significantly upregulated following AC3 deletion in the DRGs, and AC1 inhibitor NB001 completely reversed the increased cAMP level, enhanced neuronal excitability, and decreased *I*_A_ Kv current and behavioral hyperalgesia induced by *AC3* CKO. Thus, the increase in AC1 may be an alternative explanation for cAMP upregulation and hyperalgesia caused by AC3 deficiency in the DRGs. As mentioned above, AC1 has been demonstrated to be involved in nociceptive transmission. *AC1* KO or *AC1/AC8* DKO mice exhibit robustly reduced formalin and CFA inflammatory pain responses (14). Systemic administration of AC1 inhibitor antagonized neuropathic, inflammatory, and cancer pain (17, 58). Studies have shown that AC1 deletion causes significant reduction in allodynia while AC8 deletion alone has no effect, suggesting that AC1 may contribute more than AC8 to cAMP production after injury or that AC1 may functionally compensate the loss of AC8 (14, 19). Understandably, AC1 increased in a compensable manner with the loss of AC3 in the DRGs (Supplemental Figure 7). However, it remains elusive how the deficiency of AC3 alters the expression of AC1 and whether AC8 also participates in the functional compensation; these aspects need to be investigated in future studies.

### AC3 in DRGs is required for KOR-mediated analgesia.

All 3 opioid receptors, MOR, DOR, and KOR, are identified in the DRGs and involved in the response to nociception (59). These receptors are members of the GPCR family, coupled with PTX-sensitive inhibitory Gα_i/o_ proteins, and subsequently regulate AC, Ca^2+^ channels, and mitogen-activated protein kinases (60). Multiple studies have demonstrated that AC and cAMP are involved in pain- and opioid-induced analgesia (36, 39, 40, 61). In *AC1*, *AC8*, or *AC1/AC8* DKO mice, AC activator forskolin-induced nociception was reduced (15), and morphine tolerance was blunted (29, 62). In addition, blockade of MOR constitutive activity (MOR_CA_) unmasks a silent AC1 central sensitization pathway that results in hyperalgesic priming (18). In *AC5-*KO mice, the behavioral effects such as analgesia of MOR and DOR, but not KOR agonists, were lost (63). These results suggest that the various isoforms of AC may mediate different opioid receptor signaling. Our current study revealed that AC3 ablation in the DRGs completely prevented KOR agonist–induced analgesia but did not affect MOR and DOR agonist–induced analgesia. Previous studies have proved that opioid receptor agonist–mediated analgesia via inducing G-protein–coupled inwardly rectifying potassium (GIRK) currents through Gβγ protein (64, 65). However, GIRK channels were reported to be absent in mouse peripheral sensory neurons (66). Thus, other potassium channels including the Ca^2+^/cAMP-sensitive potassium channel may contribute to the analgesia of KOR. A previous study showed that KOR activation of PTX-sensitive G proteins leads to Gα_i/o_ inhibition of AC production of cAMP (67). It has been reported that activation of cAMP/PKA pathways contributes to inhibition of *I*_A_ (68) and *I*_K_ (69) in mouse neurons. The present study further showed that KOR agonist Dyn–induced increases in *I*_A_ and *I*_K_ currents of DRG neurons were completely blocked by Gα_i/o_ inhibitor PTX or ablation AC3 in DRG neurons, suggesting that classical Gα_i/o_ signaling mediated the inhibition of KOR agonists on Kv channels (Supplemental Figure 7). In addition, our present study revealed a direct interaction of KOR and AC3 proteins by PLA, Co-IP, and protein-protein docking strategy, suggesting that KOR may inhibit the catalytic activity of AC3 directly through nonclassical protein-protein interaction. Moreover, peripherally restricted KOR agonist ICI204,488 failed to relieve capsaicin-induced nociceptive behaviors in *AC3*-CKO mice, confirming the effect of DRG AC3 in peripheral KOR analgesia. It has been reported that about two-thirds of KOR-cre labeled sensory neurons are nociceptive neurons expressing CGRP or CGRP and NF200 (40). The distribution pattern of KOR in DRG neurons is similar to *AC3* mRNA distribution. These data provide cytological evidence for the regulation of KOR analgesia by DRG AC3.

In summary, the present study reveals an important role of peripheral AC3 in maintaining a basal nociceptive threshold and mediating KOR analgesia. The changes in Kv channels and excitability of DRG neurons might be the main cellular mechanism. This present study provides a strategy targeting specific AC isoforms peripherally for development of novel analgesic therapeutics.

## Methods

### Experimental animals.

Adult C57BL/6 (6–8 weeks) WT and *AC3^fl/fl^* mice (gift from Daniel R. Storm, Department of Pharmacology, University of Washington School of Medicine, Seattle, Washington, USA) were used in this study. All animals were maintained on a 12:12-hour light/dark cycle in a room with temperature of 22°C ± 1°C with food and water ad libitum. After the experiments, the animals were euthanized via carbon dioxide inhalation. All the behavioral tests and electrophysiological recordings were performed by experimenters who were blinded to the treatments.

### Reagents and drugs administration.

DAMGO (MOR agonist, catalog E7384), [D-Ala2]-deltorphin II (DOR agonist, catalog T0675), NB001 (AC1 inhibitor, catalog SML0060), PTX (catalog P9452), and capsaicin (catalog M2028) were purchased from MilliporeSigma. ICI204,488 hydrochloride (KOR agonist, catalog 0822) and Dyn (KOR agonist, catalog 3195) were purchased from Tocris Biosciences. Nalfurafine hydrochloride (KOR agonist, catalog A12579) was purchased from Adooq Bioscience. Capsaicin was dissolved in 0.01M PBS with Tween 80 and ethanol, and the others were dissolved in normal saline (NS) as stock solutions. All the stock solutions were stored at –20°C or –80°C until use. DAMGO (15 ng), [D-Ala2]-deltorphin II (5 μg), Nalfurafine hydrochloride (0.5 μg), and NB001 (2.5 μg) were delivered into the spinal space via lumbar puncture performed with 31-gauge needle between the L5 and L6 vertebrae with 5 μL volume according to previous studies (39, 40). KOR agonist ICI204,488 hydrochloride (10 mg/kg) were applied peripherally (i.p.) with 0.5–0.6 mL through i.p. injection (40). Capsaicin (0.1%) and formalin (2.5%) were injected into plantar with a 31-gauge needle.

### Preparation of acutely isolated DRG neurons.

Mice were anesthetized with isoflurane and then rapidly decapitated. The DRG tissues from spinal L3 and L4 segments were removed under microscope and immediately transferred onto DMEM (Invitrogen) on ice. The minced DRGs were treated with DMEM solution containing trypsin I from bovine pancreas (1 mg/mL, MilliporeSigma, catalog T8003) and collagenase from clostridium histolyticum (2.67 mg/mL, MilliporeSigma; catalog C9891) at 37°C for 25–30 minutes. After washing with a standard external solution, the ganglia were then gently triturated using fine fire-polished Pasteur pipettes. The isolated DRG neurons were placed onto glass coverslips in standard external solution at room temperature (RT) for 2 hours. The external solution contained (in mM) NaCl 140, KCl 5, MgCl_2_·6H_2_O 1, CaCl_2_·2H_2_O 2.5, HEPES 10, and D-glucose 10, adjusting PH to 7.2 with NaOH.

### Preparation of spinal cord slices.

The L3–L5 lumbar spinal cord segments were rapidly removed under anesthesia and transferred to ice cold cutting ACSF containing (in mM) NMDG 92, KCl 2.5, HEPES 20, NaHCO_3_ 30, Glucose 25, Na-ascorbate 5, Na-pyruvate 3, Thiourea 2, MgSO_4_ 10, and CaCl_2_ 0.5, oxygenated with 95% O_2_ and 5% CO_2_, adjusting PH to 7.3 with HCl (all from MilliporeSigma). Transverse slices (300 μm) were cut on a vibrating blade microtome (Leica VT1200S) and incubated in recording ACSF oxygenated with 95% O_2_ and 5% CO_2_ for at least 30 minutes at 32°C before recording. Slices were then transferred to the chamber and perfused with recording solution at a rate of 2.5 mL/min at RT. The recording ACSF contained (in mM) NaCl 125, KCl 2.5, NaHCO_3_ 26, NaH_2_PO_4_·2H_2_O 1.25, CaCl_2_ 2, MgCl_2_·6H_2_O 1, and Glucose 25, adjusting PH to 7.3 with HCl (all from MilliporeSigma).

### Whole-cell patch clamp recording.

Whole-cell patch clamp recording in EGFP-fluorescent labeled DRG neurons was performed at RT (23°C ± 1°C). Axonpatch 200B amplifier and a Digidata 1440A digitizer (Axon Instruments) were used. Microelectrodes were made from borosilicate capillaries bearing filament (World Precision Instruments Inc.) with P-1000 pipette puller (Sutter Instruments). The resistance of the microelectrodes was 3–5 MΩ, filled with pipette solution. After sealing cells with 1–10 GΩ, whole cell configuration was formed; then, cell membrane capacitance and series resistance were compensated over 80%. Leak current subtraction was performed using the online P/4 protocol. The data were sampled at 10 kHz and low pass–filtered at 2 kHz with pClamp (version 10.6; Axon Instrument) and were analyzed with Clampfit (version 10.2). APs were elicited by injecting a series of depolarizing currents from –10 to 160 pA in 10 pA increments with 500 ms duration in current clamp. The bath solution was standard external solution as above. The pipette solution contained (in mM) KCl 140, MgCl_2_·H_2_O 1, EGTA 5, HEPES 10, ATP·Na_2_ 3, and Na_2_·GTP 0.2, adjusting PH to 7.2 with KOH. For the recording of Kv currents, different voltage gradients were applied in voltage clamp mode. The bath solution contained (in mM) Choline Chloride 140, KCl 5, CaCl_2_ 2, MgCl_2_·6H_2_O 1, HEPES 10, CdCl_2_ 1, and D-glucose 10, adjusting PH to 7.4 with KOH. When adding 5 mM 4-aminopyridine (4-AP) or 25 mM tetraethylammonium (TEA) for differentiating potassium currents components, Choline Chloride was adjusted to 135 mM and 115 mM, respectively. The pipette solution contained (in mM) potassium gluconate 120, KCl 20, CaCl_2_ 1, MgCl_2_·H_2_O 2, EGTA 10, HEPES 10, and ATP·Na_2_ 5, adjusting PH to 7.2 with KOH. Drugs were puffed by the drug delivery system (ALA-VM8; ALA Scientific Instruments). In spinal cord slices, the whole-cell patch clamp recordings were made from lamina II_o_ neurons in voltage clamp mode. For sEPSC and sIPSC recordings, pipette solution contained (in mM) Cs-methanesulfonate 127.5, CsCl 7.5, MgCl_2_·6H_2_O 2.5, EGTA 0.6, HEPES 10, Na_2_Phosphocreatine·4H_2_O 10, Na_2_-ATP 4, and Na-GTP 0.4, adjusting PH to 7.2 with CsOH. After establishing the whole-cell configuration, neurons were held at –70 mV to record sEPSCs and at 10 mV to record sIPSCs.

### IHC/immunocytochemistry.

Mice were deeply anesthetized with urethane (1.5 g/kg, i.p.) and transcardially perfused with NS, followed by 4% paraformaldehyde (PFA, 4°C) in 0.1M PB. The L3-L5 DRGs, L3–L5 spinal cord segments, and glabrous plantar skin were removed and postfixed in the same fixative for at least 6 hours at 4°C and then dehydrated in gradient concentration sucrose at 4°C. Tissues were cut into 14 μm (DRG), 30 μm (spinal cord), and 14 μm (glabrous plantar skin) sections in a cryostat (Leica Microsystems), mounted onto superfrost plus microscope slides (Fisher Scientific) or floated in PBS for use. The sections were blocked with PBS containing 8% donkey serum and 0.3% Triton X-100 for 2 hours at RT and then incubated for 48 h at 4°C with a mixture of rabbit anti-AC3 (1:1000; catalog sc-588, Santa Cruz Biotechnology Inc.) together with mouse anti-NeuN (1:2000; catalog MAB377, MilliporeSigma), mouse anti-CGRP (1:500; catalog C7113, MilliporeSigma), IB4 (1:500; catalog 1213141, Invitrogen), mouse anti-PKCγ (1:500; catalog sc-166451, Santa Cruz Biotechnology Inc.), and guinea pig anti-TRPV1 (1:500; catalog AGP-118, Alomone Lab). The sections were washed with 0.01M PBS for 15 minutes, 3 times, and were further incubated with a mixture of donkey anti–mouse Alexa Fluor 488 (1:500; catalog A-21202, Invitrogen), donkey anti–rabbit Alexa Fluor 546 (1:500; catalog A10040, Invitrogen), and donkey anti–guinea pig Alexa Fluor 647 (1:500; catalog A-21450, Invitrogen) conjugated secondary antibodies at RT for 2 hours. For immunocytochemistry, acutely isolated DRG cells or cultured DRG neurons were grown on glass coverslips coated with Poly-D-Lysine and fixed by 4% PFA at RT for 10 minutes. The neurons were incubated overnight at 4°C with a mixture of rabbit anti-AC3 (1:1000; catalog sc-588, Santa Cruz Biotechnology Inc.) and mouse anti-Arl13b (1:100; catalog 75-287, NeuroMab) or mouse anti–KOR-1 (1:500 catalog sc-374479, Santa Cruz Biotechnology Inc.). The acutely isolated or cultured neurons were then incubated for 2 hours at RT with a mixture of donkey anti–mouse Alexa Fluor 488 (1:500; catalog A-21202, Invitrogen) and donkey anti–rabbit Alexa Fluor 546 (1:500; catalog A10040, Invitrogen) conjugated secondary antibodies, counterstained with DAPI (1:30000; catalog P36935, Invitrogen) or Nissl (1:200; catalog N21479, Invitrogen). The specificity of immunostaining and primary antibodies was verified by omitting the primary antibodies, by the CKO mice, and also by ISH. The stained sections were observed and images captured with a confocal laser-scanning microscope (Model FV1000, Olympus).

### Fluorescent ISH with RNAscope and double staining with IHC.

ISH was performed using RNAscope system (Advanced Cell Diagnostics) according to the manufacturer’s protocol. The mounting slices were pretreated with hydrogen peroxide and protease provided by RNAscope multiplex fluorescent reagent kit v2 assay (Advanced Cell Diagnostics). Hybridized samples with targeted *AC3* mRNA detected probes (Advanced Cell Diagnostics) for 2 hours. Slices were further incubated with AMP1, AMP2, or AMP3 successively for 15–30 minutes; HRP-C1, HRP-C2, or HRP-C3 for 15 minutes; Opal Dye 570 (1:1500; catalog FP1488001KT, PerkinElmer), Opal Dye 520 (1:1500; catalog FP1487001KT, PerkinElmer), or Opal Dye 690 (1:1500; catalog FP1497001KT, PerkinElmer) for 30 minutes; and HRP blocker for 15 minutes. All the procedures were proceeded at 40°C in hybridization oven. After ISH, some slices were labeled with mouse anti-CGRP antibody (1:500), IB4 (1:500), mouse anti-NF200 antibody (1:500; catalog N0142, MilliporeSigma), and rabbit anti–tyrosine hydroxylase (1:1000; catalog ab137869, Abcam) and counterstained with DAPI (1:30000) and Nissl (1:200). We evaluated samples with Olympus fluorescence confocal microscope (Olympus FV1000). Semiquantitative ACD scoring for the RNAscope assay was done as previously reported (70). The score criteria are as follows: 0 indicates no staing or < 1 dot/10 cells; 1 indicates 1–3 dots/cell; 2 indicates 4–9 dots/cell and none or very few dot clusters; 3 indicates 10–15 dots/cell and/or < 10% dots are in clusters; and 4 indicates > 15 dots/cell and/or > 10% dots are in clusters. Data are calculated as a Histo score (H-score) with the following equation: H-score = Σ (ACD score × percentage of cells per bin).

### PLA application.

PLA experiment protocol was proceeded based on the manual provided with the PLA signal detection kit (Duolink In Situ Red Starter Kit Mouse/Rabbit; catalog DUO92101, MilliporeSigma). DRG sections or isolated DRG neurons were fixed with 4% PFA and deposited on the glass slides. Samples were blocked with blocking solution in a heated humidity chamber for 1 hour at 37°C. Rabbit anti-AC3 antibody (1:1000; catalog sc-588, Santa Cruz Biotechnology) and mouse anti–KOR-1 antibody (1:500; catalog sc-374479, Santa Cruz Biotechnology) were then added to target samples at 4°C overnight. After washing with wash buffer, samples were incubated with PLA probe for 60 minutes at 37°C. Ligase and polymerase were incubated, respectively, for 30 minutes and 100 minutes at 37°C. The slides were mounted using PLA mounting medium with DAPI, and signals were detected with Olympus fluorescence confocal microscope (Olympus FV1000). All reagents employed were provided by the Duolink PLA kit.

### Western blotting.

L3 and L4 DRGs were maintained in RIPA lysis buffer (catalog 20-188, MilliporeSigma) containing PMSF (catalog 10837091001, MilliporeSigma), protease inhibitor cocktail tablets (catalog 04693116001, Roche), Na_3_VO_4_ (catalog S6508, MilliporeSigma), and NaF (catalog S7920, MilliporeSigma) for 30 minutes on ice and then were homogenized within ultrasonic cell disruption system (Branson ultrasonics corporation). Equal amounts of protein samples were separated in 8% SDS-PAGE gel (Beyotime Biotechnology) and transferred onto a PVDF blot (MilliporeSigma). Blots were blocked with 5% nonfat milk in Tris-buffer saline and incubated with rabbit anti-AC3 antibody (1:1000; catalog ab125093, Abcam), rabbit anti-AC1 antibody (1:500; catalog ab69597, Abcam), rabbit anti-Kv1.4 antibody (1:200; catalog APC-007, Alomone Labs), rabbit anti-Kv3.4 antibody (1:200; catalog APC-019, Alomone Labs), rabbit anti-Kv4.3 antibody (1:200; catalog APC-017, Alomone Labs), and goat anti–rabbit GAPDH-HRP antibody (1:10000; catalog KC-5G5, KangChen Bio-tech) at 4°C overnight. The blots were further incubated with goat anti–rabbit IgG HRP (1:5000; catalog 111-035-003, Jackson ImmunoResearch) at RT for 2 hours. We incubated the blots with Supersignal West Femto Maximum Sensitivity Substrate (catalog 34095, Thermo Fisher Scientific) and detected signals by ChemiDoc XRS+ imaging system (Bio-Rad). The intensity of specific bands was displayed and analyzed through ImageJ (NIH).

### Co-IP assay.

The DRGs were collected in reagents containing 300 mM NaCl, 50 mM Tris-HCl, 4 mM PMSF, 2 mM Na_3_VO_4_, 10 mM NaF, 1% Triton X-100, and 10% glycerol. The pretreated tissues were then homogenized within ultrasonic cell disruption system. The lysates were rotated with protein G sepharose (1:5; catalog 17-0618-01, GE Healthcare Bio-science AB) and pretreated with mouse anti–KOR-1 antibody (1 μg; catalog sc-374479, Santa Cruz Biotechnology) or normal mouse IgG (1:500; catalog sc-2025, Santa Cruz Biotechnology) for negative control at 4°C overnight. Obtained lysates were blotted with rabbit anti-AC3 antibody (1:1000; catalog ab125093, Abcam) as processed by Western blot protocol.

### Protein docking strategy.

Discovery studio and Discovery Studio’s ZDOCK module were used to perform homology modeling of AC3 sequences based on 1ab8.pdb and 1azs.pdb, and protein-protein docking with KOR (4djh.pdb).

### cAMP concentration analysis.

All the procedures were in accordance with the manufacturer’s protocol provided by cAMP Enzyme Immunoassay Kit (catalog CA201, MilliporeSigma). Protein lysates were extracted from L3 and L4 DRGs as above. Assay buffer, standards, blue cAMP-Alkaline phosphatase conjugate, yellow cAMP EIA antibody, and protein samples were added into the 96-well plates coated with antigen maintained at RT for a 2-hour reaction. The optical density (OD) was detected under 405 nm after adding substrate solution and stop solution. Data were analyzed according to nonlinear regression (curve fit) through a weighted 4-parameter logistic curve fitting program.

### Intra-DRG injection of AAV.

Mice were anesthetized with sodium pentobarbital (50 mg/kg, i.p.). Intra-DRG injection was exerted according to the previous study. Proper incision was made to expose L3 and L4 segments of vertebral column, which were then fixed on the stereotaxic instrument (Stoelting). Hemi-laminectomy were performed on unilateral side of mice, and corresponding L3 and L4 DRGs were exposed according to the previous study. The virus pAOV-CAG-EGFP-2A (AAV2/8; titer: 1.63 × 10^13^ vector genomes [V.G.]/mL, control) and pAOV-CAG-EGFP-T2A-Cre (titer: 1.43 × 10^13^ V.G./mL, Obio Technology) were injected through a glass microelectrode attached to a microinjection pump (Hamilton, Nanoliter 2010 injector, World Precision Instruments Inc.). The glass microelectrode was adjusted at about 45° to the vertebral column and inserted into L3 and L4 DRG separately. Two sites were injected into each DRG with the depth of 0.2–0.3 mm and a flow rate of 30 nL/min. The total injection volume was 300 nL per DRG. After the end of infusion, a microelectrode injection needle was maintained 5 minutes before withdrawal for the virus to diffuse sufficiently. The incision was closed with 4-0 suture. Mice were allowed to recover in a warm temperature before being sent back to their home cage. Behaviors and acute DRG dissociation were performed 2–4 weeks after virus infection. Mice that showed any surgery-related neurological deficits were excluded from the experiment. At the end of the experiments, the DRGs were sectioned to verify the virus transfection and the KO efficiency.

### Behavioral testing.

Mice were habituated to the testing environment daily for at least 3 days before testing. The von Frey test for static mechanical stimulation was assessed by measuring PWTs in response to a series of calibrated filaments ranging from 0.04 to 1.4 g (0.04, 0.07, 0.16, 0.4, 0.6, 1.0, 1.4 g). Mice were placed individually in chambers on an elevated mental mesh floor to acclimate for 30 minutes. Filaments were applied perpendicularly on the plantar surface of the hind paw with ascending order, and each was maintained for 2–3 seconds. Each filament was stimulated 5 times with a 10-second interval. The PWTs were marked as the testing value of calibrated filaments when the hind paw of a mouse was withdrawn at least 3 out of 5 times, from stimulation. The paw withdrawal frequencies were calculated through the withdrawal response frequency in a 5-time stimulation with different filaments.

Dynamic mechanical allodynia was measured by light stroking stimulation (velocity ~2 cm/s) of the lateral plantar region of the hind paw in the direction from heel to toe with a paintbrush (#0, G1220 Marie’s) (71). The test was repeated 10 times at intervals of at least 3 minutes. For each test, no evoked movement was scored as 0, sustained lifting (more than 2 seconds) of the stimulated paw or a single gentle flinching of the stimulated paw scored as 1, and one strong lateral lifting above the level of the body or a startle-like jumping scored as 2. For each mouse, the average score of 10 tests was used to indicate touch score.

Hargreaves test for thermal pain was assessed by measuring PWLs in response to a radiant heat source (IITC/Life Science Instruments). Mice were placed individually into Plexiglas chambers on an elevated glass surface and allowed to acclimate for 30 minutes. The PWLs were recorded once the mouse hind paw withdrew from the heat. The heat was maintained at a constant intensity, which produced a stable basal PWL of 9–14 seconds. A 20-second cut-off time was used for preventing tissue damage in the absence of a response.

A hot plate test for thermal pain was assessed by measuring licking duration and flinching times at 2 different temperatures of the hot plate: 52°C and 55°C (IITC/Life Science Instruments).

A pinch test for mechanical pain was assessed with a mental clip clamping on the plantar surface of mice for 1 minute. The duration of licking, biting, and flinching was recorded and assessed according to the video recorder.

Capsaicin (0.1%, 20 μL) and formalin (2.5%, 20 μL) tests were performed by i.pl. injecting unilaterally into the hind paws of mice. Mice were placed individually into Plexiglas chambers after i.pl. injection. The time spent lifting, flinching, and licking the affected paw during each 5 minutes was recorded with a video recorder.

### Statistics.

All data were presented as mean ± SEM and analyzed with Graphpad Prism 8.0 software. No statistical power calculation was conducted before the study. The sample sizes were based on our previous knowledge and experience with this design. There were no missing data. All data from different groups were verified for normality and homogeneity of variance using Kolmogorov-Smirnov and Brown-Forsythe tests before analysis. Behavioral data, electrophysiological recording, and Western blot data were analyzed using 2-tailed Student’s *t* test when comparing 2 groups or using 1-way ANOVA followed by post hoc Dunnett’s test or 2-way RM ANOVA followed by post hoc Bonferroni’s multiple-comparison test when comparing more than 2 groups. No data were excluded from statistical analysis due to outlier status. All the hypothesis testing was 2 tailed, with *P* value less than 0.05 considered statistically significant.

### Study approval.

All the animal experimental procedures were approved by the Committee on the Use of Animal Experiments of Fudan University (permit no. SYXK 2009-0082) and in accordance with policies on the use of laboratory animals issued by the International Association for the Study of Pain (IASP, Washington, D.C., USA).

## Author contributions

WWZ performed all the experiments included in this study. YL and XJF performed protein-protein docking strategy. WWZ, YL, XJF, and YQZ analyzed the data and interpreted the results. HC participated in discussion of the results. WWZ and YQZ wrote the manuscript. YQZ supervised the project.

## Supplementary Material

Supplemental data

## Version 1 12/16/2021

In-Press Preview

## Version 2 02/08/2022

Electronic publication
