# Supplementary material for: Peripheral ablation of type III adenylyl cyclase induces hyperalgesia and eliminates KOR-mediated analgesia in mice
Source: JCI Insight. 2022 Feb 8;7(3):e153191. doi: 10.1172/jci.insight.153191 (PMC8855833; doi:10.1172/jci.insight.153191)

Supplemental Information

Peripheral ablation of type III adenylyl cyclase induces hyperalgesia and eliminates KOR-mediated analgesia in mice

Wen-Wen Zhang[1], Hong Cao[1], Yang Li[2], Xian-Jun Fu[3], Yu-Qiu Zhang[1*]

[1]State Key Laboratory of Medical Neurobiology and MOE Frontiers Center for Brain Science, Department of Translational Neuroscience, Jing'an District Centre Hospital of Shanghai, Institutes of Brain Science, Fudan University, Shanghai 200032, China

[2]College of Intelligence and Information Engineering, Shandong University of Traditional Chinese Medicine, Jinan, Shandong 250355, China

[3]Qingdao Academy of Chinese Medical Science, Shandong University of Traditional Chinese Medicine, Qingdao, Shandong 260000, China

* Correspondence: yuqiuzhang@fudan.edu.cn

Inventory of Supplementary Information

Supplementary Figure 1. related to Figure 1, Photomicrographs showing the distribution of AC3 in peripheral and spinal cord.

Supplementary Figure 2. related to Figure 2, The procedure of DRG injection in $AC3^{flox/flox}$ mice**.**

Supplementary Figure 3. related to Figure 3, The excitability of EGFP negative

DRG neurons.

Supplementary Figure 4. related to Figure 3, Voltage-gated potassium (Kv) channel currents and Kv subunits expression in the DRG.

Supplementary Figure 5. related to Figure 3, TRPV1 currents is amplified in *AC3*CKO DRG neurons.

Supplementary Figure 6. related to Figure 8, Protein interaction between AC1 and KOR and predicted docking protein structure of AC3 and KOR.

Supplementary Figure 7. related to Discussion, A schematic showing how *AC3*CKO modulates neuronal excitability and mediates KOR analgesia.

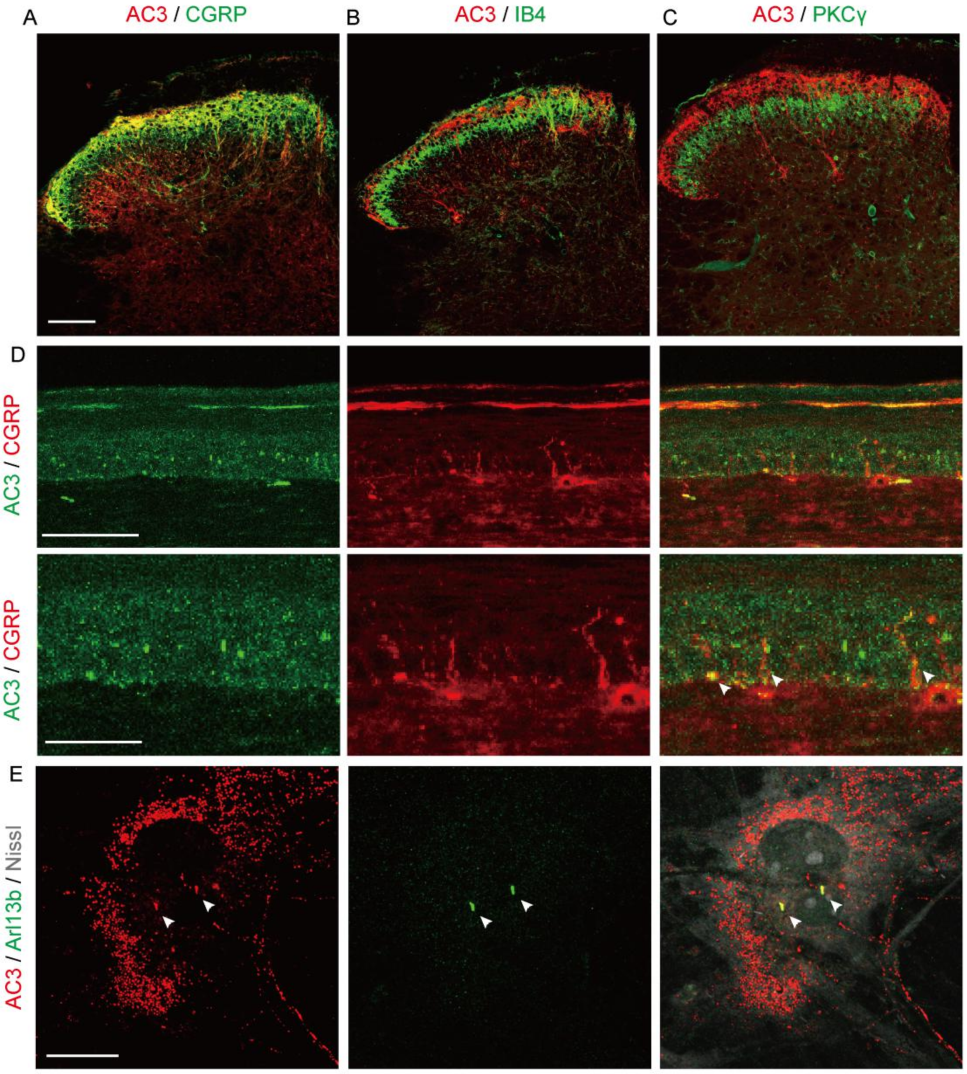

Supplementary Figure 1. related to Figure 1, Photomicrographs showing the distribution of AC3 in pheripheral and spinal cord. (**A-C**) Immunofluorescence double labeling of AC3 with CGRP- (A) and IB4-positive primary afferent terminals (B) and PKCγ-positive neurons (C) in superficial spinal dorsal horn. Scale bar: 100 µm. **(D)** Immunofluorescence double labeling of AC3 with CGRP-positive peripheral nerve terminals in the glabrous skin of mice plantar. Scale bar: 50 µm (top) and 25 µm (bottom). **(E)** Immunofluorescence double labeling of AC3 (red) and Arl13b (primary cilia marker, green) in cultured DRG neurons. Scale bar: 20 µm. Arrowheads indicate the double labeled signals.

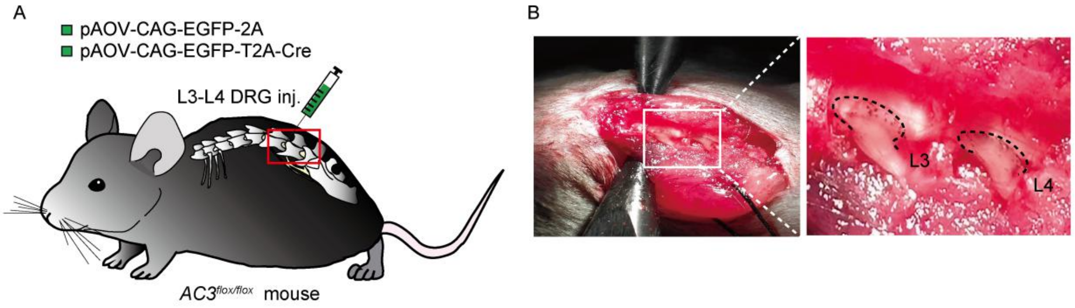

Supplementary Figure 2. related to Figure 2, The procedure of DRG injection in *AC3*$^{flox/flox}$ mice. (**A**) Schematic showing DRG injection of Cre recombinase-expressing AAV virus (pAOV-CAG-EGFP-T2A-Cre) and control AAV virus (pAOV-CAG-EGFP-2A) in *AC3*$^{flox/flox}$ mice. (**B**) Surgical field displaying the location for L3 and L4 DRG in *AC3*$^{flox/flox}$ mice.

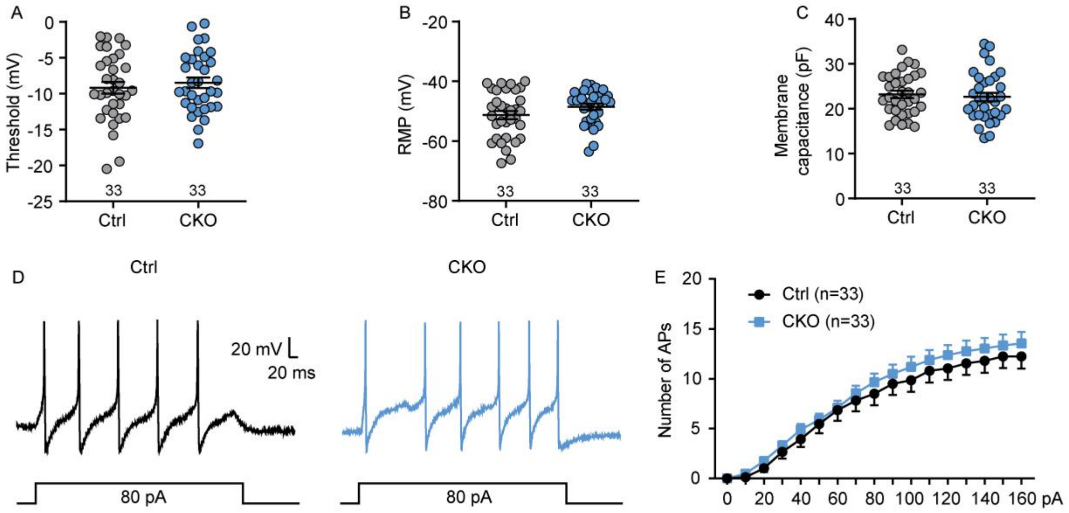

Supplementary Figure 3. related to Figure 3, The excitability of EGFP negative DRG neurons. (**A-C**) In current clamp model, EGFP negative DRG neurons from pAOV-CAG-EGFP-T2A-Cre injected mice showed similar membrane capacitance (A), resting membrane potential (B) and APs threshold (C) to that from control virus injected mice. 2-tailed Student's *t* test; n = 33 Ctrl and CKO (cells). **(D)** Examples of the AP traces of EGFP negative DRG neurons from control and Cre virus injected mice. **(E)** The numbers of APs evoked by current injection had no significant differences in DRG EGFP negative neurons between the *AC3*CKO and control mice. 2-way RM ANOVA; *n* = 33 Ctrl and CKO (cells).

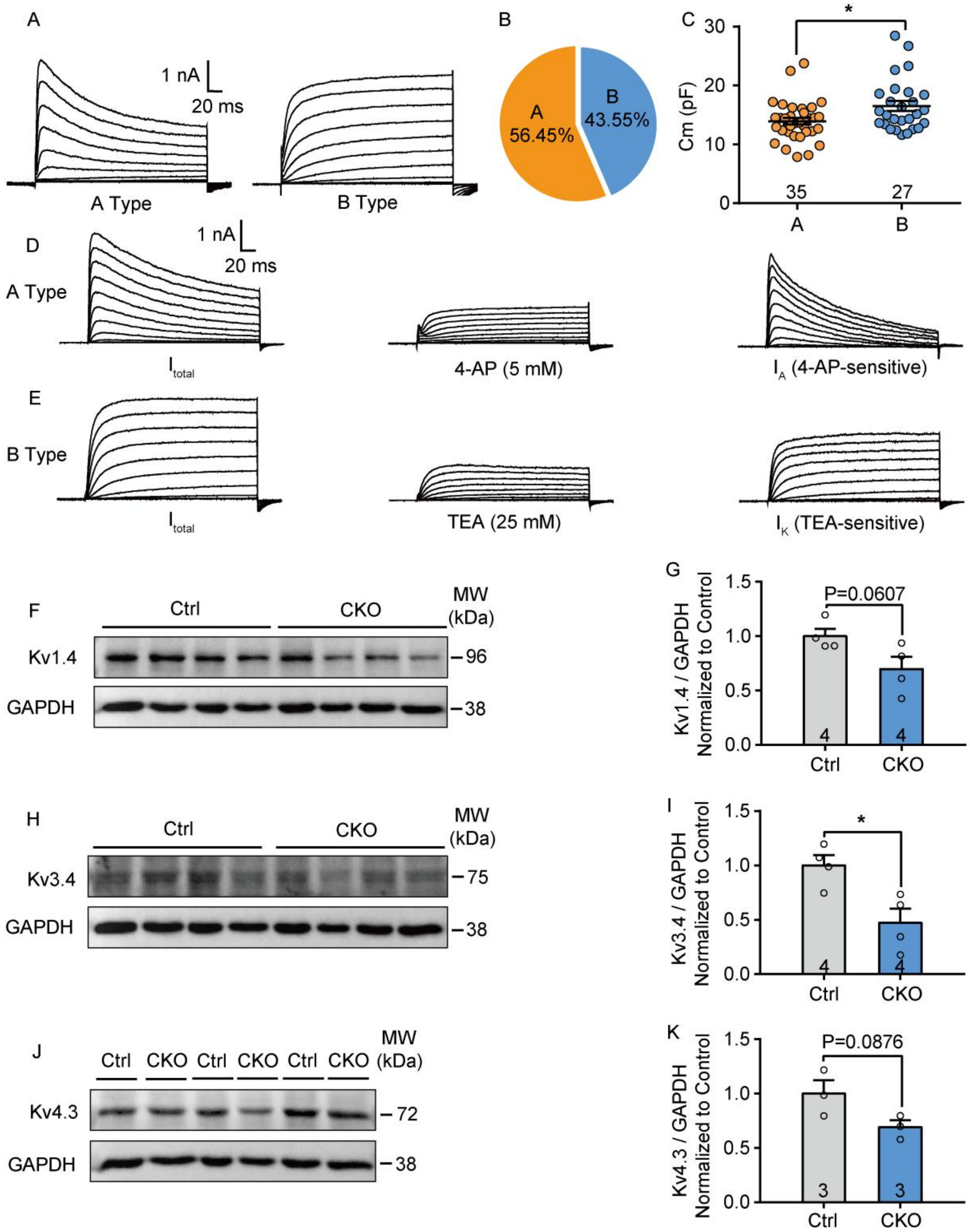

Supplementary Figure 4. related to Figure 3, Voltage-gated potassium (Kv) channel currents and Kv subunits expression in the DRG. (**A-C**) Two subpopulation of Kv channel currents including rapidly inactivating A type Kv currents and B type sustained delayed Kv currents (A) and percentage recorded for the two types (B). The membrane capacitance of A type Kv currents dominated DRG

neurons was less than that of B type Kv currents dominated DRG neurons (C). * $p < 0.05$; 2-tailed Student's t test; n = 35 A type and 27 B type (cells). (**D** and **E**) 4-aminopyridine (4-AP)-sensitive A type Kv currents (I_A) and TEA-sensitive B type Kv currents (I_K) were confirmed pharmacologically in the presence of 5 mM 4-AP (D) and 25 mM TEA (E). (**F-K**) Western blot analysis showing downregulated Kv subunits Kv1.4 (F and G), Kv3.4 (H and I) and Kv4.3 (J and K) in *AC3*CKO DRGs. Data are represented as fold changes compared with the intensity of GAPDH. * $p < 0.05$; 2-tailed Student's t test; n = 3 or 4 Ctrl and CKO (mice).

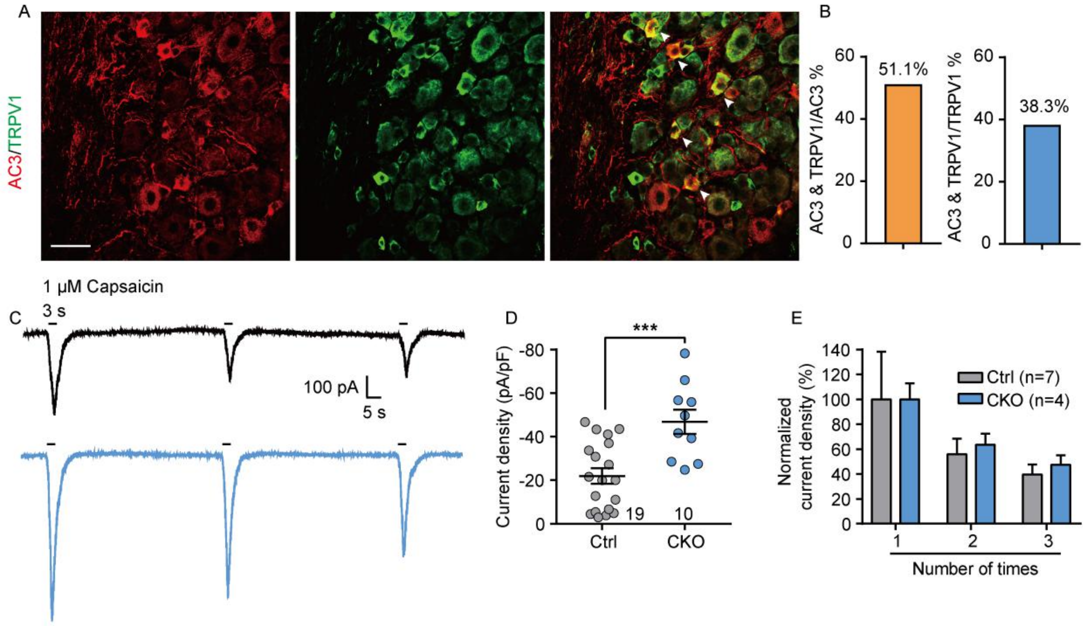

Supplementary Figure 5. related to Figure 3, TRPV1 currents is amplified in *AC3*CKO DRG neurons. (**A** and **B**) Immunofluorescence double labeling of co-localization of AC3 with TRPV1 immunoreactivities in the L4 DRG. Scale bar: 50 μm. (**C-E**) Successive applications of capsaicin (1 μM)-induced TRPV1 currents in control and *AC3*CKO DRG neurons. Repeated capsaicin-induced desensitization rate had no difference between *AC3*CKO DRG neurons and controls, but the TRPV1 current density was significantly amplified in *AC3*CKO DRG neurons comparing with controls. *** $p < 0.001$; 2-tailed Student's t test; $n = 19$ Ctrl and 10 CKO (cells).

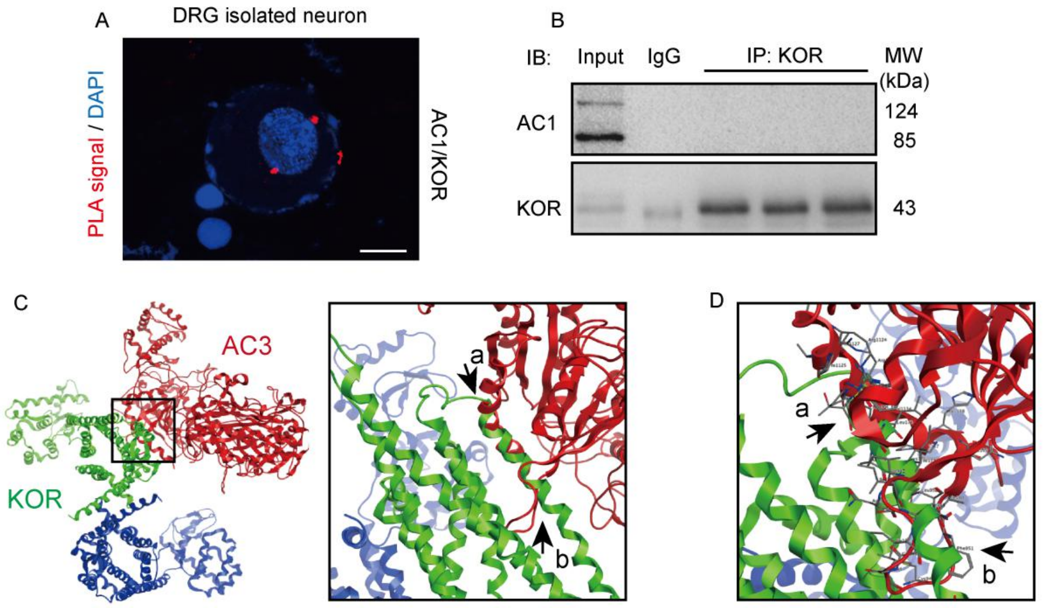

Supplementary Figure 6. related to Figure 8, Protein interaction between AC1 and KOR and predicted docking protein structure of AC3 and KOR. **(A)** Proximity ligation assay (PLA) showing sparse PLA signals of AC1 with KOR in isolated DRG neurons. Scale bar: 10 μm. **(B)** Co-immunoprecipitation displaying that AC1 (IB) cannot be captured by mouse anti-KOR antibody (IP) in mouse lumber DRGs. Normal mouse IgG immunoprecipitation was applied as the negative control. Three individual trials were performed to repeat the results. (**C** and **D**) Schematic diagram of KOR and AC3 protein-protein binding. Black arrows indicate the predicted binding structure of AC3 and KOR (C). The predicted binding protein sequence including 170Arg-196Leu α helical structure of KOR with 1123Asp-1142Asp α- helical structure (a) and 940ILE-952LEU β-turn structure (b) of AC3 (D).

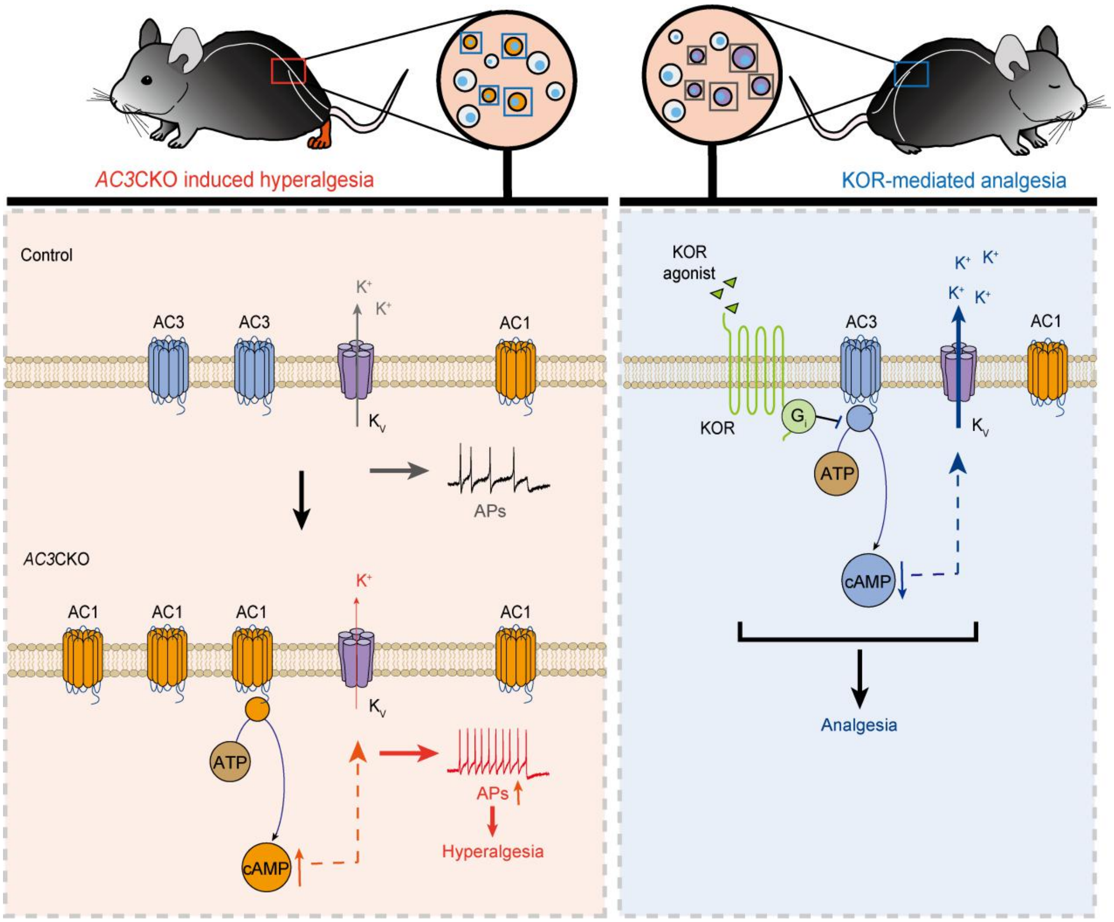

Supplementary Figure 7. related to Discussion, A schematic showing how *AC3*CKO modulates neuronal excitability and mediates KOR analgesia. Following the AC3 deletion in the DRG, AC1 might be compensatory upregulated, leading to a enhanced neuronal excitability and behavioral hyperalgesia via modulating Kv channels by cAMP. On the other hand, KOR agonist enhances the Kv currents of DRG neurons via KOR coupled Gαi protein to inhibit AC3 or directly inhibit AC3 through interaction. When AC3 deficiency, KOR-mediated analgesia faded.

Full uncut gels

Full unedited gel for Figure 2A

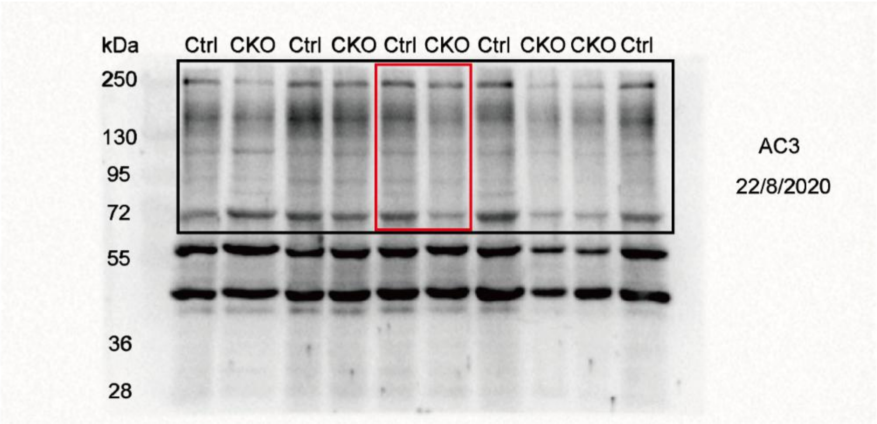

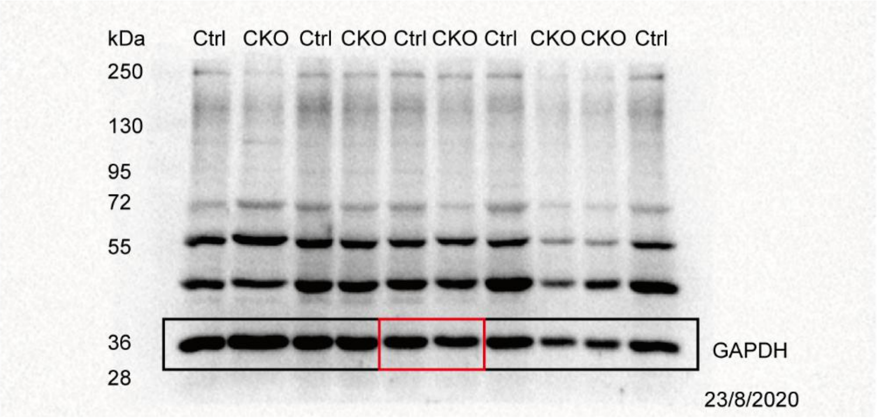

Full unedited gel for Figure 5F

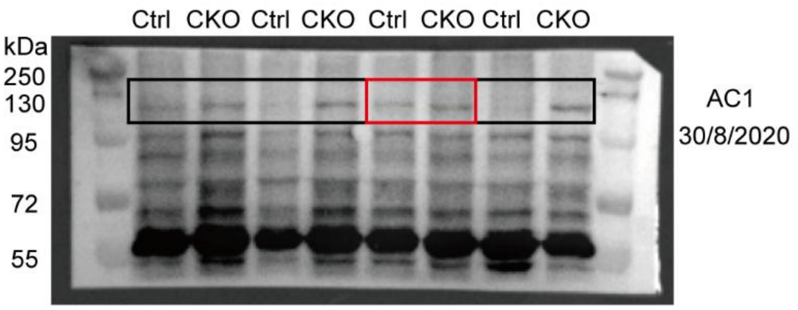

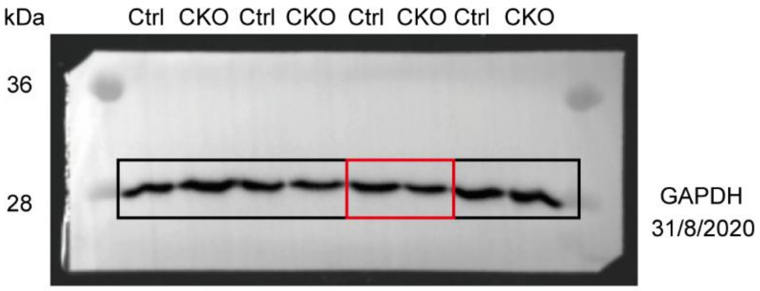

Full unedited gel for Figure 8I

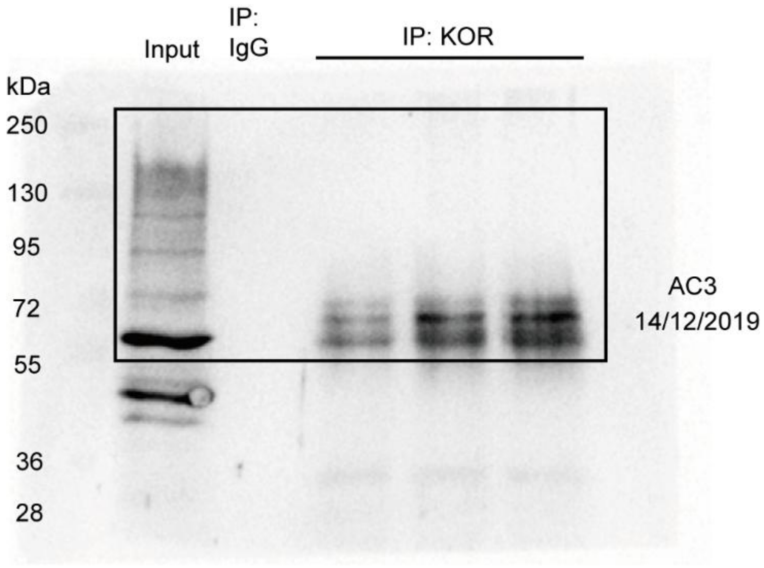

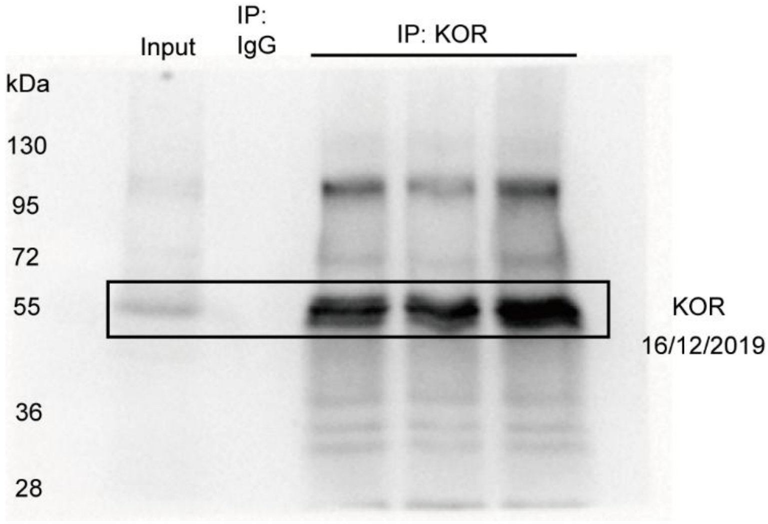

Full unedited gel for Supplementary Figure 4F

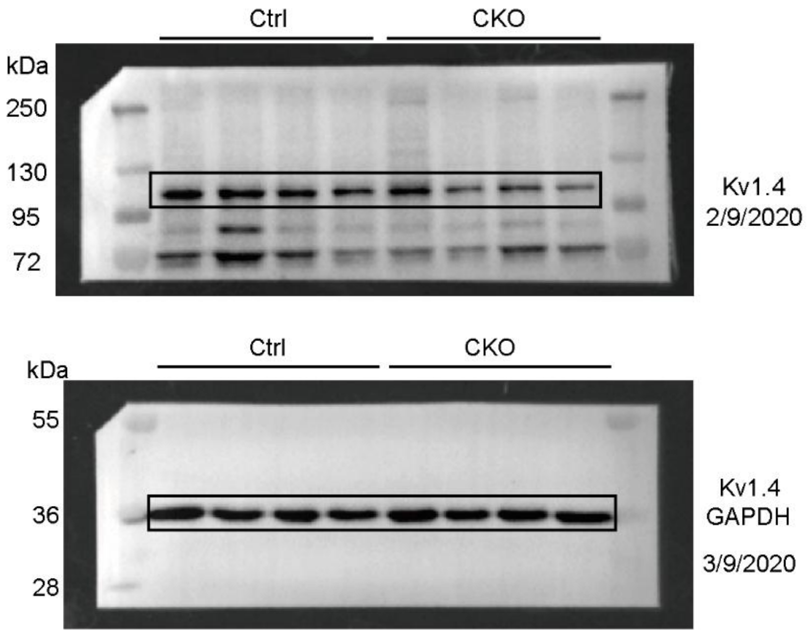

Full unedited gel for Supplementary Figure 4H

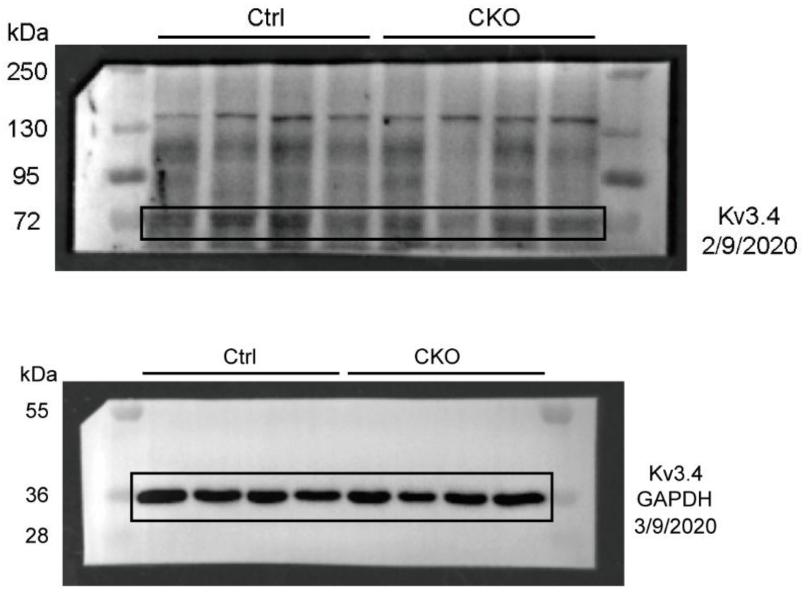

Full unedited gel for Supplementary Figure 4J

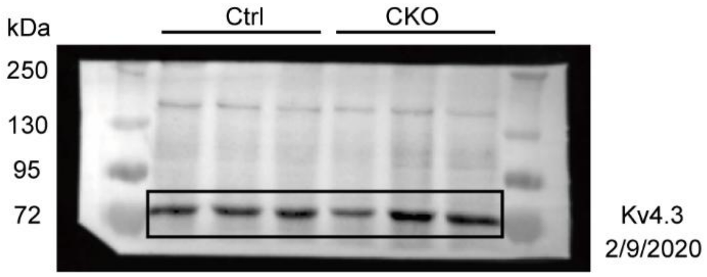

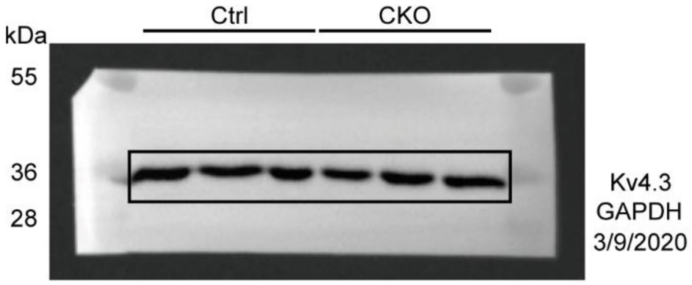

Full unedited gel for Supplementary Figure 6B

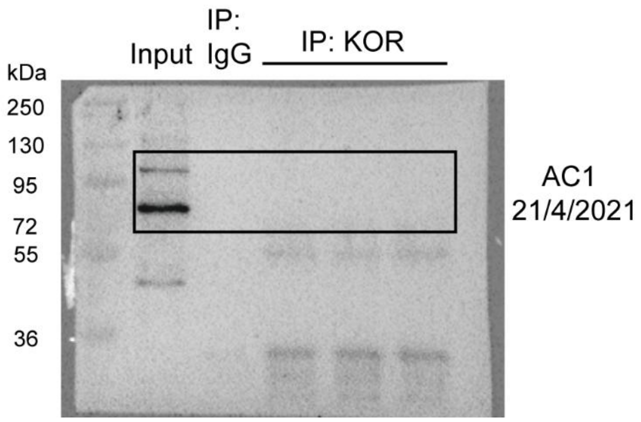

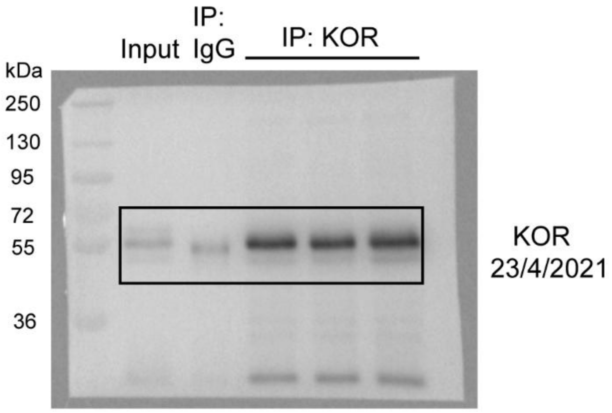

Supplement: Supplemental data [file jciinsight-7-153191-s239.pdf]
